# Kinetic and *in silico* structural characterization of norbelladine *O*-methyltransferase of Amaryllidaceae alkaloids biosynthesis

**DOI:** 10.1016/j.jbc.2024.107649

**Published:** 2024-08-08

**Authors:** Manoj Koirala, Natacha Merindol, Vahid Karimzadegan, Sarah-Eve Gélinas, Nuwan Sameera Liyanage, Basanta Lamichhane, Maria Camila García Tobón, Patrick Lagüe, Isabel Desgagné-Penix

**Affiliations:** 1 Department of Chemistry, Biochemistry and Physics, Université du Québec à Trois-Rivières, Trois-Rivières, Québec, Canada; 2 Department of Biochemistry, Microbiology and Bioinformatics, Laval University, Québec, Canada; 3 Plant Biology Research Group, Trois-Rivières, Québec, Canada

**Keywords:** Amaryllidaceae alkaloids, biosynthesis, *O-*methyltransferase, regio-selectivity, catalytic potential, molecular docking

## Abstract

Amaryllidaceae alkaloids are a diverse group of alkaloids exclusively reported from the Amaryllidaceae plant family. *In planta*, their biosynthesis is still not fully characterized; however, a labeling study established 4ˈ-*O*-methylnorbelladine as the key intermediate compound of the pathway. Previous reports have characterized *O*-methyltransferases from several Amaryllidaceae species. Nevertheless, the formation of the different *O*-methylnorbelladine derivatives (3ˈ-*O*-methylnorbelladine, 4ˈ-*O-*methylnorbelladine, and 3ˈ4ˈ-*O-*dimethylnorbelladine), the role, and the preferred substrates of *O-*methyltransferases are not clearly understood. In this study, we performed the biochemical characterization of an *O-*methyltransferase candidate from *Narcissus papyraceus* (*Np*OMT) *in vitro* and *in vivo,* following biotransformation of norbelladine in *Nicotiana benthamiana* having transient expression of *NpOMT*. Docking analysis was further used to investigate substrate preferences, as well as key interacting residues of *Np*OMT. Our study shows that *Np*OMT methylates norbelladine preferentially at the 4ˈ-OH position *in vitro* and *in planta.* Interestingly, *Np*OMT also catalyzed the synthesis of 3ˈ,4ˈ-*O-*dimethylnorbelladine from norbelladine and 4ˈ-*O-*methylnorbelladine during *in vitro* enzymatic assay. Furthermore, we show that *Np*OMT methylates 3,4-dihydroxybenzylaldehyde and caffeic acid in a nonregiospecific manner to produce meta/para monomethylated products. This study reveals a novel catalytic potential of an Amaryllidaceae *O-*methyltransferase and its ability to regioselectively methylate norbelladine in the heterologous host *N. benthamiana*.

Amaryllidaceae of the Asparagales order are herbaceous perennials plants classified into three subfamilies: *Amaryllidoideae*, *Agapanthoideae*, and *Allioideae* (1). This plant family has 75 genera and over 1600 species, which are predominantly found in subtropical to tropical climates. In addition to their ornamental qualities, *Amaryllidoidae* display potent medicinal value (2, 3). They have a long history of usage in traditional medicine. *Narcissus* species, for example, have been recognized for their potent anticancer properties since ancient times. Hippocrates of Kos, considered the father of medicine, suggested using oil extracted from *Narcissus* sp. to treat uterine tumors 400 years ago (4, 5). The medicinal properties of Amaryllidaceae plants are primarily attributed to their rich phytochemical profile, particularly the Amaryllidaceae alkaloids (AAs), which are exclusively synthesized by members of the *Amaryllidoideae* subfamily (6). Biochemical analyses have revealed that AAs are diverse in structure and pharmacological properties (7). Potent AAs include galanthamine, a specific, competitive, and reversible acetylcholinesterase inhibitor, used in the management and treatment of Alzheimer’s disease (8), as well as antiviral AAs such as lycorine, haemanthamine, and cherylline (9, 10). AAs are typically harvested from plants, for instance from *Narcissus papyraceus*, an Amaryllidaceae native to the Mediterranean region, known for its ability to thrive indoors and produce fragrant paperwhite flowers (11). Although extensive research has explored the phytochemical diversity and medicinal potential of Amaryllidaceae plants, the biosynthesis and regulatory mechanisms of AAs remain insufficiently understood, highlighting the critical need for further investigation in this area. The AAs biosynthetic pathway (Fig. 1) has been proposed based on radiolabeling experiments and partial enzyme characterization (3). All AAs derive from the aromatic amino acids _L_-phenylalanine and _L_-tyrosine, which are respectively transformed into 3,4-dihydroxybenzaldehyde (3,4-DHBA, through the synthesis of caffeic acid) and tyramine (Fig. 1). The condensation of tyramine with 3,4-DHBA or possibly with its methylated forms (isovanillin or vanillin), catalyzed by norbelladine synthase and/or norcraugsodine reductase, results in the formation of norbelladine, 3ˈ-*O*-methylnorbelladine, or 4ˈ-*O*-methylnorbelladine, respectively (12, 13, 14, 15, 16). Norbelladine was also shown to undergo 4ˈ-*O*- or 3ˈ-*O*-methylation directly (17). The step at which *O-*methylation preferentially occurs during AA biosynthesis (*i.e*. caffeic acid, 3,4-DHBA, or norbelladine) is unknown, but early radioisotope labeling studies have established 4ˈ-*O-*methylnorbelladine as a pivotal intermediate of the pathway (18, 19). On the one hand, this methylated intermediate serves as a substrate for CYP96Ts, which catalyze *para-para’*, *para-ortho’*, or *ortho-para’* couplings, leading to the formation of different core ring skeletons, including the galanthamine-, lycorine-, and crinine-type (Fig. 1) (2, 3). On the other hand, it leads to the cherylline-type through an unknown distinct enzymatic route, while the belladine ring type is thought to arise from 3ˈ,4ˈ-*O*-dimethylnorbelladine (3). Therefore, the biosynthesis of the distinct methylated forms of norbelladine includes foundational steps for structural diversification and orientation towards specific ring types in the AA pathway.

**Figure 1:**
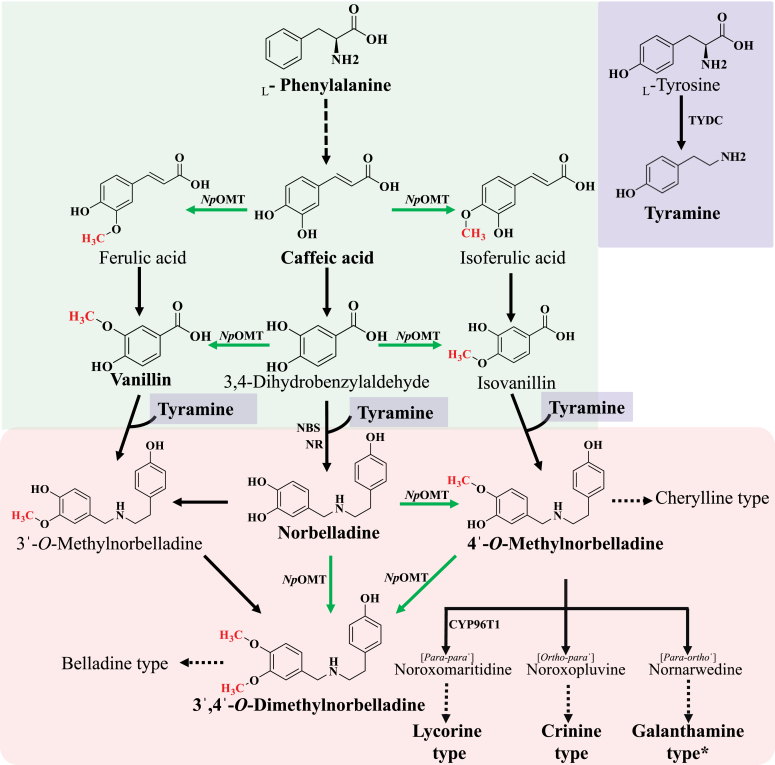
**Schematic representation of the proposed *Amaryllidaceae* alkaloid biosynthetic pathway.***Green* box, *blue* box, and *red* box represent the phenylpropanoid tyramine and amaryllidaceae alkaloid (AA) pathways, respectively. The metabolites (Amaryllidaceae precursor molecules, AAs and AA-type) written in bold were detected during this study in *Narcissus papyraceus* plant. AAs with asterisk (∗) denotes that the relative quantity of the detected metabolite was low. *Dotted* arrows indicate a series of reaction whereas *solid* arrows indicate single reaction steps. *Green* arrows indicate the reaction catalyzed by NpOMT characterized during this study.

In plant, *O-*methylation is catalyzed by *S*AM (AdoMet)-dependent *O-*methyltransferases (OMT), which transfer a methyl group from SAM to a free hydroxyl group of acceptor compounds such as alkaloids (20, 21, 22, 23). While some groups of plant OMTs exhibit specificity towards a single substrate, generally, those involved in specialized metabolites such as phenylpropanoids, flavonoids, and alkaloids accept multiple identical substrates and catalyze sequential methylation reactions (24, 25, 26). Although theoretically, *O*-methylation occur at any free hydroxyl position of a substrate, 7- and 4ˈ-methoxylation are most prevalent among flavonoids and a number of polymethoxylated flavonoids have also been reported (27). Multiple *O*-methylation steps are also involved in benzylisoquinoline alkaloid biosynthetic pathways, including at the 7-OH, 9-OH, or 2-OH positions in the reticuline, scoulerine, protoberberine, respectively (25, 28, 29).

Previous studies in Amaryllidaceae *Narcissus* sp. *aff. pseudonarcissus*, *Lycoris* sp., and *Galanthus elwesii* have shown that the *O-*methylation of different substrates of the AAs pathway is catalyzed by class I type OMTs. This class of OMT is metal-dependent and also includes catechol OMT and caffeoyl-CoA OMT (COMT and CCoAOMT, respectively). The general enzymatic process involves a direct nucleophilic attack of one of the hydroxyl groups of the catechol ring by the methyl carbon of SAM through an S_N_2-like transition state, assisted by Mg^2+^ and a lysine residue (Lys144 in COMT) (30). During the reaction, SAM binds first to the COMT, followed by the metal ion, and finally the substrate (31). Previously characterized norbelladine OMTs differ in their preference for metal cations such as Mg^2^⁺, Co^2^⁺, Mn^2^⁺, or Ca^2^⁺ (22, 32). A triple mutant of a norbelladine 4ˈ-*O-*methyltransferase from *N.* sp*. aff. pseudonarcissus* (*Nps*N4OMT)^E36P/G40E/A53M^ was recently crystallized, representing a breakthrough in the study of the AAs biosynthetic pathway (33). The resolved structure confirmed the overall COMT-like shape of the enzyme and its formation as a homodimer.

In 2014, Kilgore *et al.* enzymatically characterized *Nps*N4OMT, demonstrating that its ability to regioselectively transfer a methyl group to the free 4ˈ-OH of norbelladine and *N*-methylnorbelladine, but not to the free OH of caffeic acid and 3,4-DHBA (21). The strong regioselectivity of *Nps*N4OMT was recently confirmed (34). By contrast, homologous OMTs from *Lycoris* species were shown to catalyze methylation at the free 4ˈ or 3ˈ-OH positions of norbelladine, as well as caffeic acid and 3,4-DHBA (22, 23). Additionally, *G. elwesii* OMT can methylate diverse catechol (32), and several enzyme residues and metal ion combinations responsible for the regioselectivity were identified and confirmed through protein engineering studies. Despite these findings, the precise step at which *O-*methylation preferentially occurs (*i.e..* caffeic acid, 3,4-DHBA, or norbelladine) and the formation of dimethylated norbelladine remain unclear. Moreover, although several downstream AAs are 3ˈ,4ˈ-*O-*dimethylated, no Amaryllidaceae OMT capable of generating 3ˈ,4ˈ-O-dimethylnorbelladine has been reported.

Further characterization of Amaryllidaceae OMTs is necessary to uncover enzymes with distinct substrate preferences and understand the molecular mechanisms governing their specificity. Such investigations are pertinent to both fundamental inquiries, such as elucidating the sequence of the AAs pathway and to biotechnological endeavors. OMT-catalyzed methylation for the production of bioactive compounds is known to be more selective and requires milder conditions than chemical synthesis (35).

During this study, a candidate *NpOMT* transcript was identified and isolated from the transcriptome of *N. papyraceus*. *In silico* and biochemical investigations confirmed that *Np*OMT belongs to class I type metal-dependent OMT. *In vitro* experiments demonstrated that *Np*OMT methylated norbelladine, yielding 4ˈ-*O-*methylnorbelladine, but not 3ˈ-*O-*methylnorbelladine. The enzyme also catalyzed the synthesis of 3ˈ,4ˈ-*O-*dimethylnorbelladine from norbelladine (as minor product) and from 4ˈ-*O-*methylnorbelladine. Furthermore, *Np*OMT methylated free 3- or 4-OH of 3,4-DHBA and caffeic acid. Interestingly, *Np*OMT exhibited a higher affinity for norbelladine than other substrates, as evidenced by docking analysis and enzyme kinetics study. The enzyme’s promiscuity and its ability to methylate substrates in regioselective and nonregioselective manners can be leveraged to design specific synthetic biological approaches to rewire AAs pathway in heterologous chassis, aiming to produce specific AAs (36, 37, 38).

## Results

### *N. papyraceus* leaves are enriched in AAs, while its roots contain precursors

**Figure 2:**
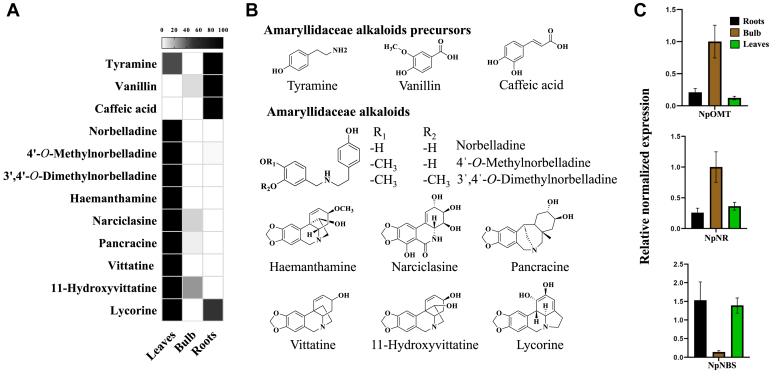
**Targeted metabolites profiling and expression of *Amaryllidaceae* alkaloids biosynthesis genes.***A*, heat map showing the organ-specific profile of targeted metabolites (AAs precursor and AAs) in *Narcissus papyraceus*. Relative abundance corresponds to the mean value of three independent replicates. Values were normalized to the sample with the highest level (100%) for each compound. *B*, structures corresponding to detected target metabolites. *C*, qRT-PCR analysis of *NpOMT*, *NpNR*, and *NpOMT* in leaves, bulb, and roots *of N. papyraceus*. *Histone3* specific primers were used as base for relative expression. Three biological and two technical replicates were considered for each gene evaluated.

To understand AAs biosynthetic capacity of *N. papyraceus,* we performed combined targeted profilings of metabolites and transcripts in the vegetative roots, bulbs, and leaves using high-performance liquid chromatograph (HPLC)-tandem mass spectrometer (MS/MS) and RT-qPCR. Overall, three AAs precursor molecules (tyramine, vanillin, and caffeic acid) and nine AAs (norbelladine, 4ˈ-*O-*methylnorbelladine, 3ˈ,4ˈ-*O-*dimethylnorbelladine, haemanthamine, narciclasine, pancracine, crinine, 11-hydroxyvittaine, and lycorine) were detected in this study (Figs 2 and S1). We observed that AAs precursor molecules accumulated at higher levels in the roots. Specifically, the levels of tyramine were 1.5 and 16.2-fold higher in roots than leaves and bulbs, respectively. Vanillin (3-*O*-methylated 3,4-DHBA) levels were 8.6-fold higher in roots than bulbs and were not detected in leaves. Isovanillin (4-*O*-methylated 3,4-DHBA) was not detected. Caffeic acid was exclusively detected in roots, while its methylated forms, that is, ferulic acid and isoferulic acid were not detected. In contrast, AAs accumulated at relatively higher levels in the leaves of *N. papyraceus*. Norbelladine, 3ˈ,4ˈ-*O*-dimethylnorbelladine, and crinine were exclusively detected in leaves, whereas 4ˈ-*O-*methylnorbelladine, narciclasine, pancracine, 11- hydroxyvittatine, and lycorine were detected in all tissues.

Then, we assessed the expression profile of candidate transcripts encoding the AA pathway foundation enzymes, *OMT*, *norbelladine synthase*, and *norcraugsodine reductase* (*i.e.*. *NpOMT*, *NpNBS*, and *NpNR*), in the same tissues (Fig. 2*C*). Putative transcripts were identified from the transcriptomic data based on their amino acid sequence identity with the characterized OMT from *N.* sp. *aff. pseudonarcissus* (39). Interestingly, gene expression levels were similar for *NpOMT* and *NpNR*, with both showing higher expression in bulbs, where vanillin and some AAs were detected, compared to leaves and roots. In contrast, the expression pattern of *NpNBS* was inverted, with the highest levels in roots, which also contained higher quantities of precursors, and in leaves where most of the alkaloids, including norbelladine and 4ˈ-*O-*methylnorbelladine, were found. Hence, *N. papyraceus* tissues presented a unique profile of unmethylated, methylated, and dimethylated products and a strong expression of the putative *NpOMT* in bulbs.

### *N. papyraceus O-*methyltransferase candidate is a class I type metal-dependent OMT

**Figure 3:**
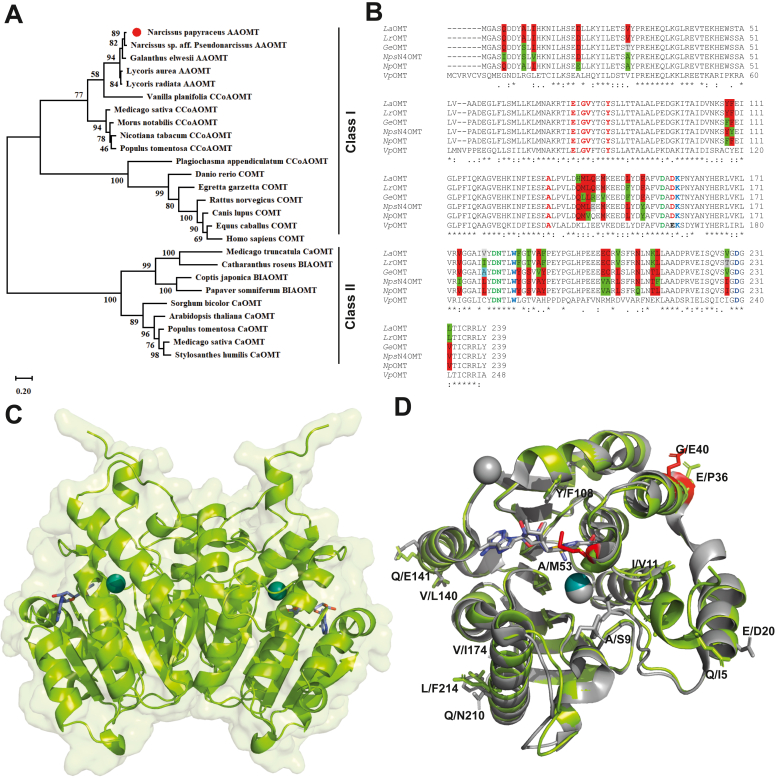
**Phylogenetic analysis, multiple sequence alignment, and predicted 3D model of *Np*OMT.***A*, phylogenetic tree compare evolutionary relation of *Np*OMT with other characterized plant OMTs. The phylogenetic tree was constructed using the MEGA 6.0 software package and neighbor-joining program (http://www.megasoftware.net) with 1000 bootstrapped value support. The amino acid residue sequences of the plant OMT were obtained using the National Center for Biotechnology Information (NCBI) search engine (http://www.ncbi.nlm.nih.gov/protein/). The accession numbers are provided in Table S2. Amino acid sequence of *Lycoris aurea* AAOMT (*La*OMT) was isolated from reference (23). The scale indicates the evolutionary distance. *B*, multiple amino acid residue sequence alignment was performed with *Np*OMT, *L. radiata* OMT (*Lr*OMT), *La*OMT, *N.* sp. *aff pseudonarcissus* OMT (*Np*N4OMT), and *Vanilla panifolia* OMT (*Vp*OMT) by using cluster omega online tool (https://www.ebi.ac.uk/Tools/msa/clustalo/). The highly conserved amino acids involved in interactions with SAM are highlighted in *red*. The highly conserved amino acids that contribute to metal ion binding are marked in *green*, and the residues involved in the catalysis of the methyl transfer are highlighted in *yellow*. *C*, predicted structure of *Np*OMT homodimer (AF-A0A346TLF5-F1, in *light green* cartoon with transparent surface), with SAM (as *purple* sticks) and Mg^2+^ (as *blue* sphere) in the binding pocket incorporated using PDB:1H1D coordinates. *D*, superimposition of *Np*OMT (in *light green*) and crystalized *Nps*N4OMT^E36P/G40E/A53M^ variant (in *gray*, PDB:8UKE). Variable residues between the two proteins are shown as sticks; induced mutations (E36P/G40E/A53M) are displayed in *red*. AAOMT, Amaryllidaceae Alkaloid *O-*methyltransferase; BIAOMT:, benzylisoquinoline alkaloid *O-*methyltransferase; CaOMT, caffeic acid *O*-methyltransferase; CCoAOMT, caffeoyl CoA *O-*methyltransferase; COMT, catechol *O-*methyltransferase.

The *NpOMT* sequence was subject to more in-depth investigation. The candidate *NpOMT* has 720 nucleotides and translation products displayed an expected molecular mass of 27.16 kDa with 239 amino acids and a predicted isoelectric point of 4.87. Phylogenetic analysis revealed that *Np*OMT clusters together with other Amaryllidaceae alkaloid OMT (AAOMT) in a single clade of class I type of metal dependent OMT. This clade is also closely related to CCoAOMT from *Vanillin planifolia* (*Vp*OMT) (Fig. 3*A*, Table S1). The class I OMT clade also encompassed COMT, while the distinct class II OMT clade included caffeic acid (CaOMT) and benzylisoquinoline *O-*methyltransferase. This result was consistent with characterized norbelladine *O-*methyltransferases from other Amaryllidaceae species (21, 22, 23). Multiple sequence alignment further revealed that *Np*OMT shared 95.40%, 92.05%, 91.63% and 92.89% amino acid sequence identity with characterized norbelladine OMT from *N.*sp *aff*. *pseudonarcissus* (*Nps*N4OMT), *Lycoris radiata* (*Lr*OMT), *Lycoris aurea* (*La*OMT), and *G. elwesii* (*Ge*OMT) (Table S2, Fig. 3*B*). *Np*OMT shows a high conservation in the amino acid sequences involved in SAM binding (in red, Val55, Glu77, Gly79, Val80, Tyr84, Ala132, and Asp157), metal binding (in green, Asp155, Asp181 and Asn182), and methyl transferase catalysis (Lys158, Trp185, and Asp230) (Fig. 3*B*). Noteworthy, Tyr186 is replaced by Phe186 in both *La* and *Lr*OMT (Table S3).

To determine the three-dimensional folding of the *Np*OMT dimer, we analyzed the predicted model generated by AlphaFold and assembled it as a homodimer (Fig. 3*C*). The enzyme exhibited structural similarities to other COMT structures, each monomer comprising seven β strands primarily constituted by hydrophobic residues in its core, surrounded by a succession of α-helices and loops on the protein’s surface (Fig. 3, *C* and *D*). Each *Np*OMT monomer, like other COMT, contains a single catalytic pocket, consisting of a Rossman-like fold that binds the cofactor SAM and is oriented outward, facing the exterior of the protein. Both SAM and Mg^2+^ were incorporated into predicted structure upon superimposition of active site residues with reported COMT crystal structures (PDB:1H1D, Fig. 3*C*). Mg^2+^ interacted with Asp155 (n = 2), Asp181, Asn182, while SAM formed hydrophobic bond with Tyr81, H-bonds with Val55, Tyr81, Tyr84, Ser85, Asp103, (n = 2), Val104, Ala132, Asp155, Asp157 (n = 2), and a salt bridge with Asp155 (Table S4), consistently with the identified conserved residues of class I CCoAOMT (Fig. 3*B*). We compared the predicted structure of *Np*OMT to the resolved crystal of the *Nps*N4ˈOMT^E36P/G40E/A53M^ variant (RMSD = 0.437). The overall 3D structures closely matched each other, although a dissimilarity in the folding of the N-terminal loop could be noted. Of note, this region was modeled with less confidence by AlphaFold (Fig. S2, *A* and *B*). There were also differences in residues surrounding the active site Gln/Ile5, Ala/Ser9, Ile/Val11, Ala/Met53 (introduced by mutagenesis), Tyr/Phe108, but also at the exterior surface of the enzyme, including Glu/Asp20, Glu/Pro36 (mutagenesis), Gly/Glu40 (mutagenesis), Val/Leu140, Gln/Glu141, Val/Iso174, Gln/Asn210, Leu/Phe214 (Fig. 3*D*). We compared predicted structure of *Np*OMT and *Lr*OMT (Fig. S2*C*) and observed that the structures were highly similar (RMSD = 0.074), with differences mainly in the N-terminal region. Hence, the structural analysis confirms that *Np*OMT resembles enzymes capable of catalyzing metal-dependent *O-*methylation of norbelladine and also displays differences in sequence and structure compared to previously characterized norbelladine OMT, which could lead to distinct substrate specificity and regioselectivity.

### *Np*OMT is a norbelladine 4ˈ-*O-*methyl and 3ˈ4ˈ-*O-*dimethyltransferase

To gain insight into the catalytic activity, recombinant *NpOMT* was expressed and its enzymatic activity assessed using norbelladine. Targeted ORF of *NpOMT* transcript was amplified from bulb cDNA, and the recombinant N-terminal-MBP-tagged *Np*OMT enzyme expressed in *Escherichia coli* was purified using amylose-resin chromatography. The purity of *Np*OMT was assessed using SDS-PAGE (Fig. S3). The molecular weight of the purified protein and the predicted translation products were similar (approximately 72 kDa with MBP-tag).

**Figure 4:**
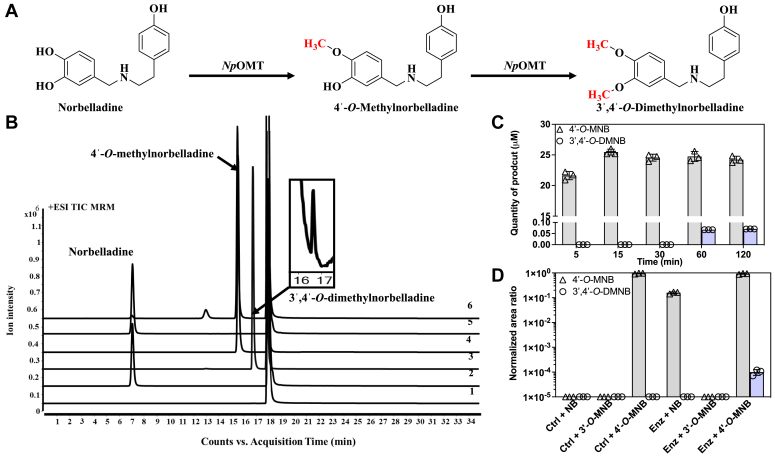
**LC-MS/MS analysis of *in vitro* enzymatic assay with norbelladine.***A*, schematic conversion of norbelladine into 4ˈ-*O-*methylnorbelladine and 3ˈ,4ˈ-*O-*dimethylnorbelladine. *B*, + ESI TIC MRM chromatogram with detection of assay products in MRM mode. (1) No substrate, (2) No SAM, (3) standard 3ˈ-*O-*methylnorbelladine, (4) standard 4ˈ-*O-*methylnorbelladine, (5) enzymatic assay with heat-deactivated *Np*OMT, and (6) enzymatic assay with *Np*OMT. *C*, enzymatic assay of *Np*OMT with norbelladine at 5, 15, 30, 60, and 120 min. All experimental values represent the means of three independent replicates. *D*, enzymatic assay of *Np*OMT with norbelladine (NB), 3ˈ-*O-*methylnorbelladine (3ˈ-*O-*MNB), or 4ˈ-*O-*methylnorbelladine (4ˈ-*O-*MNB) as substrates (all at 100 μM), measuring the production of 4ˈ-*O-*methylnorbelladine and 3ˈ4ˈ-*O-*dimethylnorbelladine (3ˈ4ˈ-*O-*DMNB) following 30 min incubation. Ctrl are negative controls that include 1) corresponding substrate and SAM without enzyme, 2) heat-activated enzyme with SAM and substrate, and 3) enzyme with substrate without SAM. A background noise (a signal corresponding to 3ˈ,4ˈ-*O-*dimethylnorbelladine) was detected when using 3ˈ-*O-*methylnorbelladine as substrate and was subtracted to all values using this substrate. All experimental values represent the means of three replicates.

Norbelladine displays three free OH-groups associated with a catechol or a phenol ring at C4ˈ, C3ˈ, and C4 positions that could theoretically be *O-*methylated. During incubation with *Np*OMT, 96 to 98% of the norbelladine substrate was converted into reaction products and the LC-MS/MS analysis revealed the presence of three new product peaks at retention time 13.10 min, 15.63 min, and 16.67 min (Fig. 4, *A* and *B*), with an increase of 14 Da for peaks at 13.10 min and 15.63 min and of 28 Da for the peak 16.67 compared to norbelladine molecular mass. This suggested that mono- and di-methylated products of norbelladine were formed. The major product at 15.63 min was confirmed as 4ˈ-*O*-methylnorbelladine ([M + H]^+^ ion *m/z* 274), while the minor peak at 16.67 min was identified as 3ˈ,4ˈ-*O*-dimethylnorbelladine ([M + H]^+^ ion *m/z* 288), by comparison with available authentic standards (Fig. S4). The peak (iv) displayed an [M + H]^+^ ion *m/z* 274 but its fragmentation pattern and parent ions did not match neither 4ˈ-*O*- nor 3ˈ-*O*-methylated norbelladine (Figs. 4*B* and S4*B*). The reaction product ion mass matched with monomethylated norbelladine at 4-hydroxyl position; however, this could not be confirmed, as the authenticated standard of 4-*O*-methylnorbelladine was not available.

To understand the formation of 3ˈ,4ˈ-*O*-dimethylnorbelladine, we performed an enzymatic reaction with norbelladine in 5 to 120 min time series (Fig. 4*C*). This showed exclusive formation of 4ˈ-*O*-methylnorbelladine for the first 30 min, followed by concomitant detection of the latter together with 3ˈ,4ˈ-*O*-dimethylnorbelladine from 60 to 120 min, albeit at much lower levels. Further enzymatic reactions were performed using norbelladine, 4ˈ-*O*- and 3ˈ-*O*-methylnorbelladine as substrates. The formation of the dimethylated product was not observed when using norbelladine or 3ˈ-*O*-methylnorbelladine as substrate in this 30 min assay. However, 3ˈ,4ˈ-*O*-dimethylnorbelladine was specifically detected when 4ˈ-*O*-methylnorbelladine was added to *Np*OMT, while it remained undetected in the corresponding negative controls (Fig. 4*D*). These results confirm that the candidate *Np*OMT catalyzes 4ˈ-*O*-methylation of norbelladine and shows for the first time that *Np*OMT can also catalyze the formation of 3ˈ4ˈ-*O*-dimethylnorbelladine from 4ˈ-*O*-methylnorbelladine (Table 1).

**Table 1:** Enzyme promiscuity of NpOMT with different types of substrates

Substrate	Major product	Minor product
Norbelladine^a^	4ˈ-*O* methylnorbelladine	3ˈ,4ˈ-*O*-dimethylnorbelladine
Caffeic acid	Ferulic acid	Isoferulic acid
3,4-DHBA	Vanillin	Isovanillin
4ˈ-*O* methylnorbelladine	3ˈ,4ˈ-*O*-dimethylnorbelladine	Npd
3ˈ-*O* methylnorbelladine	Npd	Npd
Vanillin	Npd	Npd
Isovanillin	Npd	Npd
Ferulic acid	Npd	Npd
Isoferulic acid	Npd	Npd
Tyramine	Npd	Npd

Npd, no new product detected.

a An additional unknown peak was detected on the reaction mixture.

### *Np*OMT also accepts AAs precursors as substrates

**Figure 5:**
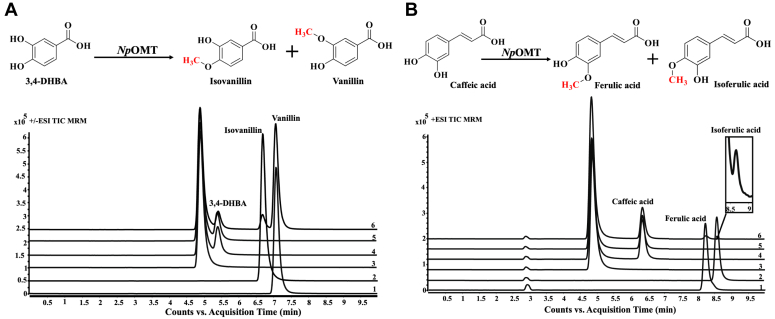
**LC-MS/MS analysis of *Np*OMT enzymatic assay with 3,4-DHBA and caffeic acid.***A*, schematic diagram and +ESI TIC MRM chromatograms of *Np*OMT-catalyzed enzymatic reaction with 3,4-DHBA. Diagram shows the formation of isovanillin and vanillin. (1) No 3,4-DHBA, (2) No SAM, (3) standard isovanillin, (4) standard vanillin, (5) no protein enzymatic reaction, (6) enzymatic assay with 3,4-DHBA. *B*, schematic diagram and +ESI TIC MRM of *Np*OMT-catalyzed enzymatic reaction with caffeic acid. Diagram shows the formation of ferulic acid and isoferulic acid. (1) No caffeic acid, (2) No SAM, (3) standard ferulic acid, (4) standard isoferulic acid, (5) no protein enzymatic reaction, (6) enzymatic assay with caffeic acid.

Subsequently, we sought to measure the enzyme ability to methylate the AAs precursor compounds 3,4-DHBA, caffeic acid, and tyramine. 3,4-DHBA and caffeic acid have two free hydroxyl groups associated with a benzene ring at C3 and C4 positions, and tyramine has a free hydroxyl group at C4 position. *Np*OMT did not catalyze the *O*-methylation of tyramine (Table 1). However, when using 3,4-DHBA or caffeic acid as substrates, two new peaks appeared (Fig. 5, *A* and *B*). The observed new peaks had an increased mass of 14 Da relative to each substrate. This indicated that methylation happened in a single position for both 3,4-DHBA or caffeic acid. Using 3,4-DHBA as substrate, 88 to 91% of substrate was converted to the reaction products. The products were confirmed as vanillin for the major product ([M + H]^+^ ion *m/z* 153, peak at 7.15 min) and isovanillin ([M + H]^+^ ion *m/z* 153, peak at 6.50 min) by comparison with authentic standards. In the case of caffeic acid, only 6 to 8% of the substrate was converted into the reaction products. The products were confirmed as ferulic acid ([M + H]+ *m/z* 195, peak at 8.10 min) for the major product and isoferulic acid ([M + H]^+^ ion *m/z* 195, peak at 8.50 min).

To test for dimethylation catalysis by *Np*OMT, enzymatic reactions were carried out using ferulic acid, isoferulic acid, vanillin, or isovanillin as substrate. No new product peak was detected in the enzymatic reactions using *Np*OMT with any of these substrates (Table 1). Altogether, these results suggest that *Np*OMT accepts norbelladine, 4ˈ-*O*-methylnorbelladine, and unmethylated phenylpropanoid precursors as substrates.

### Key differences between *Np*OMT, *Nps*N4OMT, *Lr*OMT, *La*OMT, and *Ge*OMT

Previously characterized Amaryllidaceae norbelladine OMTs were compared at sequence level to get insight of the amino acid residues governing substrate specificity (Fig. S5, Table S3). A detailed comparative analysis of the enzymes capable of catalyzing 3ˈ- and 4ˈ-*O*-methylation of norbelladine (*La*OMT and *Lr*OMT), regioselective towards norbelladine 4ˈ-*O*-methylation (*Ge*OMT, *Nps*N4ˈOMT), and catalyzing 4ˈ-*O*-methylation combined with 3ˈ,4ˈ-*O-*dimethylation (*Np*OMT), as well as their ability to methylate caffeic acid and 3,4-DHBA (*Lr*OMT, *Ge*OMT, and *Np*OMT) or not (*Nps*N4ˈOMT) revealed 19 unique residues across these scenarios (Table S3). Specifically, enzymes reported to 3ˈ-*O*-methylate norbelladine exhibited Val30, Gln141, Val/Ile179, Phe186, Thr188, Phe191, Val206, Lys213, Leu232, compared to Ala/Thr30, Glu/Arg141, Leu/Ala179, Tyr186, Ser188, Tyr191, Leu206, Thr213, Val232 in enzymes that only 4ˈ-*O*-methylated this substrate (Figs. 3 and S5). *Np*OMT that catalyze norbelladine dimethylation showed Glu20 and Gln210 as unique residues. More variability was observed with regards to the ability to *O*-methylate 3,4-DHBA and caffeic acid with ≥2 possibilities at each of these positions.

Intriguingly, many of the identified residues were exposed at the surface of the enzyme (Fig. S5), suggesting that they do not participate in direct interactions with the ligands. However, others, like Ala9, Glu20, Ala30, Phe108, Tyr148, Tyr151, Leu179, Tyr186, Ser188, Tyr191, Ile228, Val232, surrounds the catalytic pocket and could be implicated in the network supporting substrate interaction. In particular, Tyr186 (Phe186 in enzymes that catalyze the 3ˈ-*O*-methylation of norbelladine) is a key catalytic residue.

### Enzyme kinetic study of *Np*OMT with 3,4-DHBA and norbelladine

The optimal conditions for enzymatic reaction of *Np*OMT were determined by incubating *Np*OMT with 3,4-DHBA at temperatures ranging from 20 to 60 °C and pH from 4.5 to 13. Vanillin and isovanillin production were optimal at 45 °C and pH = 7.4 (Fig. S6). Metal ions have been shown to impact on class I OMT regioselectivity (32). To determine their effect on the rate of reaction, we compared product formation in the presence of EDTA, Mg^2+^, Zn^2+^, Ni^2+^, Co^2+^, Ca^2+^, or Mn^2+^, using norbelladine or 3,4-DHBA as substrates. In presence of EDTA, the reaction rate was decreased to barely detectable levels, as expected (Fig. S7, *A*–*C*). Overall, the reaction rate of norbelladine 4ˈ-*O*-methylation increased similarly upon addition of any metal ions in the reaction compared to no salt control, except for Ca^2+^, in which case, the rate decreased. These results indicated that *Np*OMT requires divalent metal ions other than Ca^2+^ to catalyze this reaction. The highest rate of reaction for the formation of isovanillin was observed in the presence of Ni^2+^ (Fig. S7*B*), while Zn^2+^ led to the highest rate of vanillin formation. This revealed that the type of metal ion impacts on the regioselective methylation of 3,4-DHBA. We further performed a kinetic study of *Np*OMT using two different metal ions Zn^2+^ and Ni^2+^ for 3,4 DHBA and Mg^2+^ for norbelladine. For the formation of vanillin, the lowest *K*_m_ value (491 ± 51 μM) was observed with Zn^2+^, but the difference was not statistically significant. In the case of isovanillin biosynthesis, the lowest *K*_*m*_ value (627 ± 77 μM) was observed with Ni^2+^(*p* = 0.05, one tailed Mann-Whitney test). *V*_max_ and *K*_cat_ values associated with the synthesis of both vanillin and isovanillin favored the presence of Ni^2+^, as compared to Zn^2+^ (Table 2).

**Table 2:** Kinetic parameters of *Np*OMT-catalyzed methylation of norbelladine and 3,4-DHBA

Substrate	Norbelladine	3,4-DHBA			
Product	4ˈ-*O*-methylnorbelladine	Vanillin		Isovanillin	
	Mg^2+^	Zn^2+^	Ni^2+^	Zn^2+^	Ni^2+^
*K*_cat_ (min^−1^)	2.17 ± 0.24	3.82 ± 0.15	4.48 ± 0.16	0.21 ± 0.01	0.35 ± 0.02
*K*_m_ (μM)	169 ± 19	491 ± 51	544 ± 58	869 ± 130	627 ± 77
*V*_max_ (μM min^−1^)	10.86 ± 1.25	19.09 ± 0.75	22.41 ± 0.81	1.07 ± 0.07	1.77 ± 0.08
*K*_cat_/*K*_m_ (mM^−1^ min^−1^)	12.82	7.81	8.30	0.24	0.56

Results are presented as mean of three replicates ± SD.

To prevent the formation of dimethylated norbelladine and concentrate the kinetic study on 4ˈ-*O*-methylnorbelladine as product, the reaction was quenched after 30 min of incubation. *Np*OMT catalys of norbelladine *O*-methylation followed the Michaelis–Menten kinetic with a *K*_m_ of 169 ± 19 μM and a *K*_cat_ of 2.17 min^−1^. The *V*_max_ and *K*_cat_*/K*_m_ values for the reaction were 10.86 ± 1.25 μM min^−1^ and 12.82 μM^−1^ min^−1^, respectively (Table 2 and Fig. S7*F*). Overall, *Np*OMT exhibited a preference for 4ˈ-*O*-methylation of norbelladine (*p* = 0.05 compared to *O*-methylation of 3,4-DHBA), followed by 3-*O*-methylation of 3,4-DHBA over its 4-*O*-methylation (*p* = 0.05 when Zn^2+^ was used).

### *Np*OMT localizes in the cytoplasm and methylates norbelladine *in planta*

**Figure 6:**
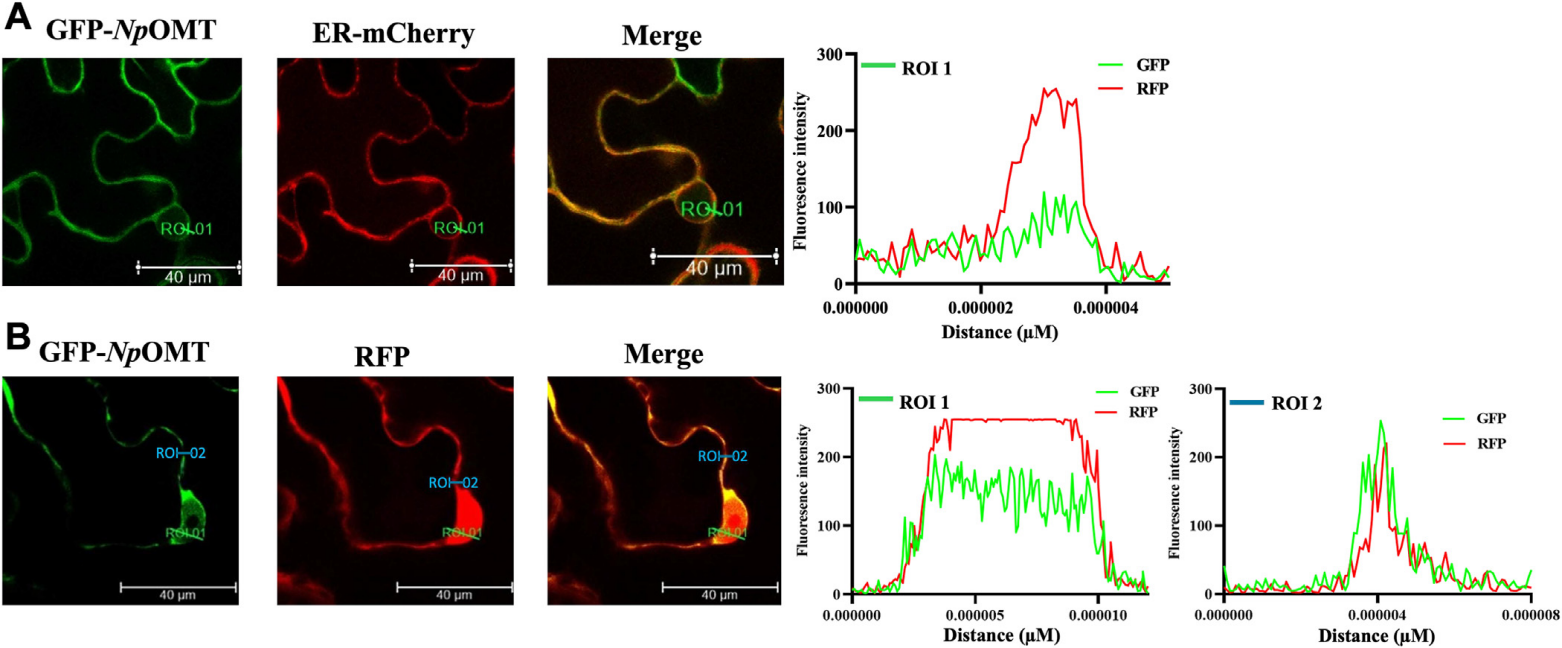
**Subcellular localization of N-terminal GFP-tagged *Np*OMT (GFP-*Np*OMT).** N-terminal GFP fusion *Np*OMT was co-expressed with *red* fluorescent protein (RFP) or ER-mCherry constructs in the epidermal cell of *Nicotiana benthamiana* leaves, and images were taken after 48 h with confocal microscopy. *A*, GFP-*Np*OMT, ER-mCherry, merge image, and graphical intensity of fluorescent intensity are shown. *B*, GFP-*Np*OMT, RFP, merge image, and graphical intensity of fluorescent intensity are shown. Scale bar represents 40 μm. ROI 1 and ROI 2 indicate fluorescent intensity in nucleus and cytosol, respectively.

To determine the subcellular localization of *Np*OMT, we used N- and C-terminal GFP-tagged *Np*OMT. The fusion proteins were identically expressed under the control of the 35S promoter in *Nicotiana benthamiana* epidermal leaf cells, with RFP as a control for cytosol and nucleus localization, and endoplasmic reticulum (ER)-mCherry as an ER-localized control. GFP-tag-*Np*OMT subcellular localization indicated a cytosolic localization, overlapping completely RFP fluorescence in the cytosol (Fig. 6). The expression patterns of C-terminal GFP-tagged *Np*OMT (*Np*OMT-GFP) were similar with GFP-*Np*OMT (Fig. S8*A*). The correct expression of the full-length GFP-tagged *Np*OMT fusion proteins was further confirmed by Western blot analysis with GFP antibodies in the *Agrobacterium*-infiltrated *N. benthamiana* leaves (Fig. S8*B*).

Norbelladine was then infiltrated into *N. benthamiana* leaves having a transient expression of GFP-*Np*OMT, as confirmed by confocal microscopy. LC-MS/MS analysis of infiltrated leaves methanolic extracts confirmed the presence of methylated norbelladine ([M + H]^+^ *m/z* 274) (Fig. S9). A comparison with authenticated standards identified the methylated product as 4ˈ-*O*-methylnorbelladine, consistently with our *in vitro* experiments. However, we did not detect the dimethylated product following the *in vivo* enzymatic assay. This indicated that *Np*OMT can regioselectively methylate norbelladine in a heterologous cellular environment.

### Differential positioning for 3ˈand 4ˈ-*O*-methylation are predicted *in silico*

To get insights into promiscuity and specificity of *Np*OMT, substrates including 3,4-DHBA, caffeic acid, norbelladine, and 4ˈ-*O*-methylnorbelladine were docked into the enzyme active site. The crystalized structure of COMT (1H1D and 3A7E) served as references for placing the metal ion and the methyl donor as well as for analyzing docking results. Initially, SAM and Mg^2+^ were positioned in the predicted active site of *Np*OMT, followed by the application of a 250 ns molecular dynamics simulation to optimize the enzyme’s conformation and its interactions with the methyl donor and metal ion. This resulted in a narrower pocket, where Mg^2+^, Lys13 from the N-terminal loop and Lys158 from the active site were stabilized by additional interactions, and SAM methyl group better oriented towards the catechol ring’s putative site (Fig. S10, Table S4). The interactions between SAM and Mg^2+^ and the active site residues largely corresponded to those of characterized enzymes (Fig. 3*B* and Table S4). The correct docking poses were selected based on the superimposition with the catechol’s ring in 1H1D, 3A7E, and of caffeoyl-CoA OMT (1SUJ) and interactions with Mg^2+^ and Lys158 positioned similarly to Lys144 in COMT (Table S4).

**Figure 7:**
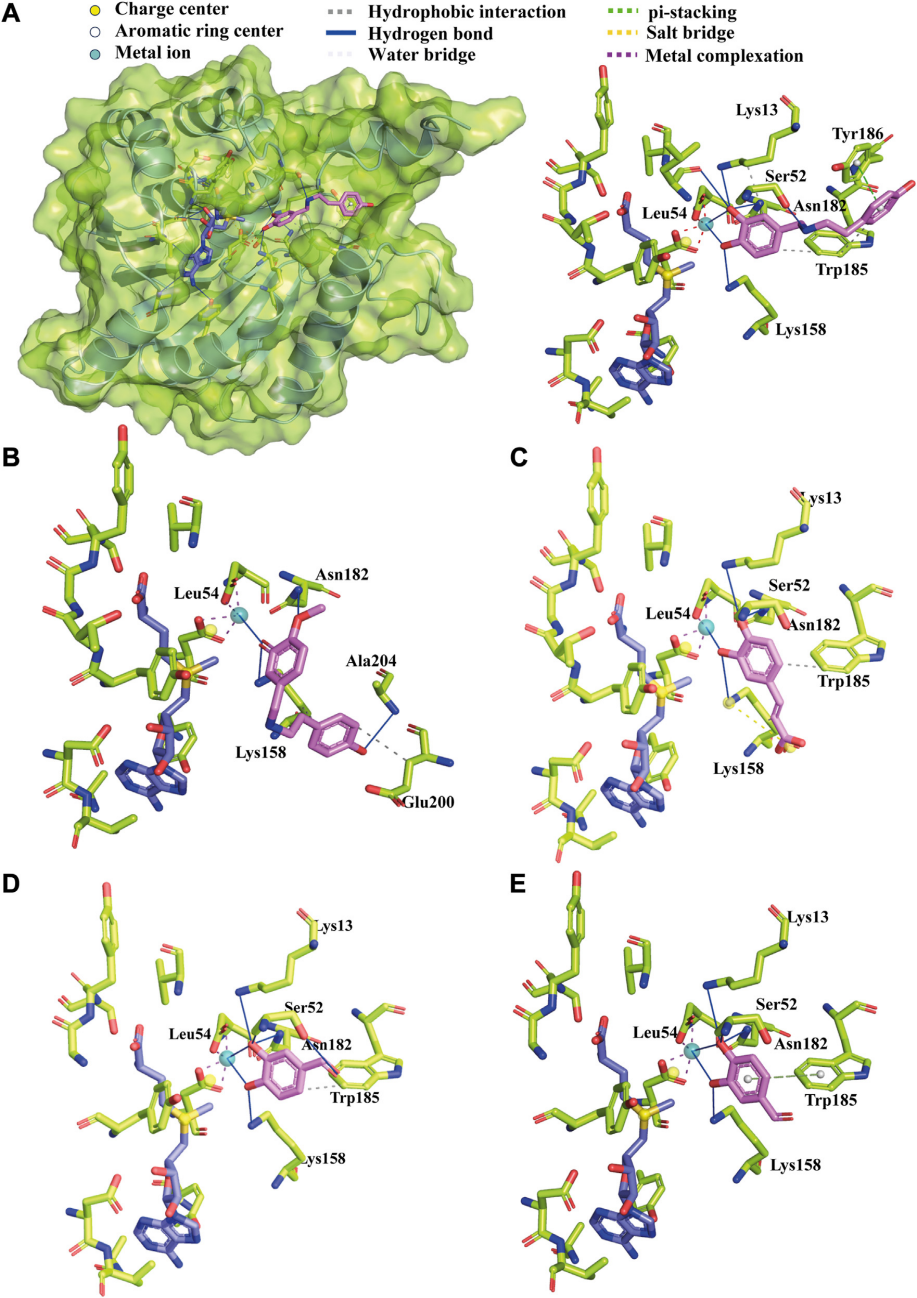
**Molecular docking of *Np*OMT with norbelladine, 4ˈ-*O*-methylnorbelladine, caffeic acid, and 3,4-DHBA.***A*, *left*: *Np*OMT (in ribbon structure overlapped that transparent surface) interacting with norbelladine (*light pink* sticks), Mg^2+^(*deep teal* sphere), and SAM (*violet* sticks). *Right*: docked norbelladine (*pink* sticks) in position for 4ˈ-*O*-methylation, SAM (*violet* sticks), and Mg^2+^ (*deep teal* sphere) interacting with *Np*OMT residues (*light green*). *B*, docked 4ˈ-*O*-methylnorbelladine (*pink* sticks) in position for 3ˈ-*O*-methylation, SAM (*violet* sticks), and Mg^2+^ (*deep teal* sphere) interacting with *Np*OMT residues (*light green*). *C*, docked caffeic acid (*pink* sticks) in position for 3-*O*-methylation, SAM (*violet* sticks), and Mg^2+^ (*deep teal* sphere) interacting with *Np*OMT residues (*light green*). *D*, docked 3,4-DHBA (*pink* sticks) in position for 4-*O*-methylation, SAM (*violet* sticks), and Mg^2+^ (*deep teal* sphere) interacting with *Np*OMT residues (*light green*). *E*, docked 3,4-DHBA (*pink* sticks) in position for 3-*O*-methylation, SAM (*violet* sticks), and Mg^2+^ (*deep teal* sphere) interacting with *Np*OMT residues (*light green*).

In the docking pose associated with 4ˈ-*O*-methylation of norbelladine, the catechol ring aligned closely with crystallized ligands reaching a docking score of −6.26 kCal/mol (Fig. 7*A*). The 3ˈand 4ˈ-OH groups of norbelladine were placed on either side of Mg^2+^, with the 4ˈ acceptor oxygen interacting with Lys158 close to the methyl donor sulfur atom of SAM (Fig. 7*A*). The 3ˈ-OH group formed interactions with Lys13, Ser52, Leu54, and Mg^2+^. The stabilization of norbelladine was further enhanced by hydrophobic interaction with Trp185 and π-stacking with Tyr186, both of which are critical catalytic residues in other OMTs (Figs 3*A* and 7*A*, Table S4).

No poses favorable to 3ˈ-*O*-methylation of norbelladine, which would align nicely with the ligands of crystallized COMT or CCoAOMT were detected. However, such pose were observed for 3ˈ-*O* deprotonated 4ˈ-*O*-methylnorbelladine with a score of −5.42 kCal/mol, albeit less optimal than the position for 4ˈ-*O*-methylation of norbelladine. By contrast to the latter, the catechol group of 4ˈ-*O*-methylnorbelladine shifted to expose its 3ˈ-O^-^ towards the methyl donor, binding to Mg^2+^ and Lys158, with Asn182 interacting with the 4ˈ-OH and Ala204 with the 4-OH (Fig. 7*B*, Table S4). The fewer interactions confirmed that this reaction was less favorable.

Caffeic acid was favorably positioned for the methylation of its 3-OH group achieving a score of −4.70 kCal/mol, while no suitable poses for 4-OH methylation were observed (Fig. 7*B*, Table S4). The 3-OH of the ligand interacted with Lys158 and Mg^2+^. Docked 3,4-DHBA was in favorable positions for a nucleophilic attack on its 4-OH with a score of −4.56 kCal/mol and on its 3-OH group with a score of −4.19 kCal/mol (Fig. 7, *C* and *D*, Table S3). The similar docking scores for these reactions with less than <1 kCal/mol differences suggest that both 3 and 4-*O*-methylation of 3,4-DHBA can occur. Notably, the ligands tended to lie parallel to SAM when positioned for 3-*O*-methylation and almost perpendicular when in position for 4-*O*-methylation. Consistent with norbelladine docking results, the ligands were stabilized by Ser52, Asn182, and Trp185. Overall, these docking analysis supports the enzymatic assay results, suggesting that norbelladine undergoes 4ˈ-*O*-methylation, 4ˈ-*O*-methylnorbelladine, 3ˈ-*O*-methylation, caffeic acid, 3-*O*-methylation, and 3,4-DHBA, both 3 and 4-*O*-methylation.

## Discussion

Although AAs are known for their diverse biological activity and wide range of structural diversity, their biosynthetic route is still not clear. The proposed biosynthesis considers norbelladine as first common intermediate compound of this pathway, in which regioselectively methylated norbelladine, such as 3ˈ-*O*-methylnorbelladine, 4ˈ-*O*-methylnorbelladine, and 3ˈ,4ˈ-*O*-dimethylnorbelladine, lead to structurally diverse end products of AAs metabolic pathway (3). Radioisotope labeling studies have established 4ˈ-*O*-methylnorbelladine as the branching point of the biosynthesis of different types of AAs, including galanthamine and lycorine (18, 19). Here, we performed a targeted metabolites analysis of *N. papyraceus* tissues, which is well known to accumulate various types of AAs, including galanthamine. Interestingly, the analysis revealed the presence of norbelladine, 4ˈ-*O*-methylnorbelladine, and 3ˈ,4ˈ-*O*-dimethylnorbelladine. The accumulation of AAs in the leaves of *N. papyraceus* rather than in other tissues during the vegetative stage was consistent with previous reports (39). The higher expression of *Np*OMT candidate in other tissues suggested that it could also methylate precursors such as 3,4-DHBA or caffeic acid. Alternatively, the uncorrelated patterns of AAs and biosynthesis enzyme transcripts could suggest that enzymes and/or alkaloids are transported to different tissues after synthesis, similar to nicotine biosynthesis and storage in tobacco plant (40).

Phylogenetic analysis identified the candidate as a class I, metal-dependent *O*-methyltransferase, clustering with CCoAOMT and COMT, while alignment confirmed the presence of the key conserved residues of the active site involved in SAM and metal binding and catalysis. Homodimer *Np*OMT candidate folding was highly similar to other characterized norbelladine-OMT, defined as class I OMT, adopting the Rossmann-like fold with an αβα sandwich structure at its core, a domain required for SAM binding (41). Class I OMT catalytic activity is dependent on the presence of a divalent metal ion like Mg^2+^, in the vicinity of SAM donor sulfur atom. The metal ion stabilizes and interacts directly with conserved active site residues Asp155, Asp181, and Asn182 and with the substrate (42, 43). On the outside of the protein, the N terminus α-helices and loops play a significant role in oligomerization and modulation of the substrate specificity (44). Interestingly, some variations were observed in that region between the *Nps*N4OMT variant crystal and the *Np*OMT predicted folding, suggesting that they may differ in catalytic potential. However, Alphafold was reported to predict less accurately this extremity of the protein (33). *Np*OMT also differed from *Nps*N4OMT, *Ge*OMT, *Lr*OMT, and *La*OMT in several residues surrounding the cavities and at the exterior surface of the enzyme. This suggested that *Np*OMT might possess a unique regioselectivity and substrate promiscuity.

Further enzymatic assays showed that *Np*OMT preferably catalyzed a metal-dependent methylation of norbelladine at 4ˈ-OH position with lower *K*_*m*_ value and higher efficiency, than reported for homologous norbelladine OMT from *Lycoris* species (22). *Lr*OMT and *La*OMT could also monomethylate norbelladine at the 3ˈ hydroxyl position, suggesting that these enzyme pockets can accomodate norbelladine in different orientations to allow both methylations (22, 23, 32). By contrast, *Np*OMT did not catalyze 3ˈ-*O*-monomethylation of norbelladine. Unlike any other reported AAOMT, *Np*OMT rather produced dimethylated norbelladine from norbelladine and 4ˈ-*O*-methylnorbelladine. The enzyme yielded an additional reaction product at a retention time of 13.1 min with a mass of [M + H]^+^ ion *m/z* 274 corresponding to a monomethylated norbelladine. However, the fragmentation pattern of product peak at 13.1 min (peak iv) did not match with neither 4ˈ-*O*- and 3ˈ-*O*-methylnorbelladine. It is possible that *Np*OMT can methylate at 4-OH position of norbelladine, but this was not confirmed, as we lack 4-*O*-methylnorbelladine as standard. *Np*OMT preference toward the 4ˈ-hydroxyl group of norbelladine could be associated with the higher accumulation of AAs derived from 4ˈ-*O*-methylnorbelladine, such as galanthamine- and lycorine-types of AAs, detected in the *in planta* screen. 3ˈ,4ˈ-*O*-dimethylnorbelladine detected *in vivo* and following enzymatic assay with *Np*OMT could lead to belladine-types of AAs, which were unfortunately not present in the current targeted AA database. Finally, contrasting to *Nps*N4OMT (21), but similarly to *Lr*OMT, *La*OMT, and *Ge*OMT, *Np*OMT methylated caffeic acid and 3,4-DHBA to produce monomethylated products at both 3- or 4-OH position. Like other mammalian and bacterial OMTs that catalyze methylation at both 3- or 4-OH position of catechol, *Np*OMT did not accept mono-methylated precursor compounds. Previous studies have highlighted the importance of the presence of two vicinal phenolic hydroxyl group in the substrate, to allow interactions with the metal ion which stabilize the substrate in the active site of the enzymes (22, 45). However, the dimethylation of 4ˈ-*O*-methylnorbelladine showed that one free OH might be sufficient for a larger ligand, although the reaction was much less favorable. Docking results suggested that deprotonation of the free OH and additional interactions with Glu200 and Ala204 may be required for correct positioning of the ligand in that situation.

The docking analysis also supported 3-*O*-methylation of 3,4-DHBA, caffeic acid, as well as 4(ˈ)-*O*-methylation catalysis of 3,4-DHBA and norbelladine by *Np*OMT. An intricate array of bounds with *Np*OMT collectively anchor the substrate molecules such that the reactive oxygen atom (at position 3 or 4) forms favorable interactions with the enzyme’s catalytic groups and SAM methyl donor. Lys158 and Mg^2+^ bound with the oxygen to be methylated, and Asn182 interacted with the other hydroxyl group of the catechol ring, emphasizing their key role in the catalytic process (46). The most stable docking pose was observed for norbelladine, involving the three characterized catalytic residues Lys158, Trp185, and Tyr186 and laying perpendicular to SAM, consistently with crystallized ligands of COMTs and CCoAOMTs. While previous reports on *Ge*OMT and (−)-epigallocatechin-3-gallate, *O*-methyltransferase from tea plant suggested that Tyr186 was required to catalyze meta (3) methylated products synthesis (32), in the case of *Np*OMT, the residue interacted with norbelladine in position favorable for 4ˈ-*O*-methylation. Other studies have described Trp185 as an important amino acid (46), here, it stabilized norbelladine, 3,4-DHBA, and caffeic acid. The recently resolved crystal structure of a triple mutant variant of *Nps*N4OMT in complex with SAH and Ca^2+^ show that Lys13 was further from the cation and closer to Tyr186 than the corresponding AlphaFold2 prediction (33). In their *in silico* analysis, upon docking norbelladine in the mutant active site, the ligand conformation was bent. This positioning contrasts with our results and with crystallized ligands in COMT and CCoAOMT structure. Several differences (natural and induced), in residues and in metal ion (Ca^2+^ *vs* Mg^2+^) in the enzyme pocket, could explain this disparity. Indeed, Ca^2+^ decreased drastically the activity of *Ge*OMT and of the original *Nps*N4OMT (21, 32). In this study, *Np*OMT activity was also blocked by the addition of Ca^2+^ instead of Mg^2+^ while using Ni^2+^ instead of Zn^2+^ modulated the *O*-methylation of some precursor compounds.

Nineteen unique residues were identified when comparing substrate promiscuity and regioselectivity of previously characterized norbelladine OMTs. Some of these residues surrounded the active site while others laid at the external surface of the protein. They could be implicated in the network supporting substrate interaction, in enzyme dimerization or in interaction with other enzymes. Enzymes, such as *Lr*OMT and *La*OMT that 3ˈ-*O*-methylate norbelladine, exhibited a unique catalytic Phe186, instead of Tyr186 in enzymes that could not. Previous studies had shown that Tyr186Lys or Tyr184Arg inverted the regioselectivity of *Ge*OMT and ECCG OMT, respectively (32, 47). *Np*OMT displayed Glu20 and Gln210 as unique residues that could correlate with its ability to catalyze norbelladine dimethylation. *Nps*4OMT and *Ge*OMT that are regioselective to 4ˈ-*O-*methylation of norbelladine had a unique variation in Glu/Arg141 instead of Gln141. A great variability in sequence was observed with regards to enzymes’ ability to *O*-methylate 3,4-DHBA and caffeic acid, suggesting high plasticity to accomodate this smaller substrate.

This study exposes that *Np*OMT is a promiscuous enzyme which can accept both AAs and precursor molecules. The promiscuous behavior *Np*OMT for 3,4-DHBA and caffeic acid suggests a possible alternative path, where methylated norbelladine would be generated from the condensation of previously methylated precursor molecules. As *Np*OMT clusters with CCoA OMT, it will be interesting to study its ability to methylate CoA substrates in future studies, possibly yielding to an alternate path to synthetize *O*-methylated norbelladine. Nonetheless, our enzymatic characterization, *in planta* investigation, and *in silico* analysis support that *Np*OMT favors the the regioselective formation of 4ˈ-*O*-methylnorbelladine *in vivo*.

## Conclusion

Overall, this study unveils a novel catalytic potential of norbelladine *O*-methyltransferase from *N. papyraceus*, predominantly yielding 4ˈ-*O*-methylnorbelladine but also 3ˈ,4ˈ-*O*-dimethylnorbelladine. Investigating the key amino acids involved in substrate interactions will enhance our understanding of AAOMT’s promiscuous behavior. Since regioselective methylation significantly influences the metabolite fate of norbelladine, gaining a more profound insight into the enzymatic catalysis mechanism is crucial. Such knowledge will aid in developing synthetic biological tools for the production of specific alkaloids in heterologous hosts.

## Experimental procedures

### Chemical reagents

Caffeic acid (98%), ferulic acid (99%), and papaverine (98%) standards were purchased from Sigma-Aldrich. 3,4-DHBA and isovanillin (98%) standards were bought from Acros Organics. Standards of 3ˈ-*O*-methylnorbelladine, 4ˈ-*O*-methylnorbelladine, 3ˈ4ˈ-*O*-dimethylnorbelladine, and norbelladine were synthetized, as described in (48). Standards of the alkaloids 11-hydroxyvittatine, haemanthamine, homolycorine, obliquine, pancracine, sanguinine, tazettine, and crinine were kindly obtained from Professors Antonio Evidente and Marco Masi (Universitario Monte Sant'Angelo). SAM was purchased from New England Biolab Inc. Standards of lycorine (98%), papaverine (98%), tyramine (99%), and vanillin (99%) were procured from Millipore Sigma. Standards of galanthamine (98%) were purchased from Tocris Bioscience. Analytical LC-MS grade methanol (99.9%) and formic acid (99%) were purchased from Thermo Fisher Scientific.

### Plant sample and metabolites extraction

*N. papyraceus* bulbs were purchased from Florissa (https://florissa.com) and were planted in autoclaved soil (ARGO MIX G6 potting soil). The plants were grown in room temperature with 14:10::light:dark condition for 18 months and were watered when necessary. Different tissues (leaves, bulb, and roots) were harvested at the vegetative stage of plant, then flash frozen under the liquid nitrogen and stored at −80 °C.

The frozen tissues (leaves, roots, and bulbs) of *N. papyraceus* were ground into powder using mortar and pestle with the aid of liquid nitrogen, and 100 mg of homogenized tissues were used for metabolite extraction. Crude metabolites extraction was done by adding 1 ml of 90% methanol then kept in the sonication bath for 1 h followed by 2 h in a 60 °C water bath. Then extracts were filtered using 0.2 μm syringe filters. Presence of targeted metabolites was detected and identified by HPLC-MS/MS.

### Identification of *Np*OMT and phylogenic analysis

To identify potential *OMT* transcript, we performed a local blast in *N. papyraceus* transcriptome by using blast 2+ software, using characterized norbelladine-*O*-methyltransferase from *N.* sp *aff. pseudonarcissus* (*Nps*N4OMT) (KJ584561) as query sequence (39). This yielded one potential OMT candidate from *N. papyraceus* (*Np*OMT) (MF979869). Protein molecular weight and pI predictions were made using the expasy tool (https://web.expasy.org/compute_pi/). Further blastx was performed in the NCBI database to compare with similar OMT. Amino acid sequence alignments were performed using the default parameters of cluster Omega (https://www.ebi.ac.uk/Tools/msa/clustalo/). Evolutionary analyses were conducted in MEGA11 (49). The evolutionary history was inferred using the neighbor-joining method (50). The tree is drawn to scale, with branch lengths in the same units as those of the evolutionary distances used to infer the phylogenetic tree. The evolutionary distances were computed using the Poisson correction method (51) and are in the units of the number of amino acid substitutions per site. This analysis which involved 25 amino acid sequences belongs to AAOMT, CCoAOMT, benzylisoquinoline alkaloid *O*-methyltransferase, and COMT. All positions containing gaps and missing data were eliminated (complete deletion option).

### RNA extraction, cDNA synthesis, and qPCR analysis

Total RNA was extracted from homogenized tissues of *N. papyraceus* (*i.e.*. leaves, bulbs, and roots) using TRIzol reagent (Invitrogen). Briefly, for 100 mg of homogenized tissues, 1 ml of TRIzol reagent was added. The liquid was transferred to a microcentrifuge tube, incubated 5 min at room temperature, and extracted with 200 μl chloroform. Following centrifugation at 12,000*g* for 15 min at 4 °C, the upper phase containing RNA was transferred to a fresh tube. The RNA was precipitated with 500 μl of isopropanol (Thermo Fisher Scientific) for 10 min at room temperature and centrifuged at 12,000*g* for 10 min at 4 °C. The RNA pellet was washed twice with 1 ml of 75% ethanol (with DEPC water) and centrifuged at 7500*g* for 5 min at 4 °C. Finally, RNA pellet was air dried and suspended in 40 μl of DEPC-treated water. The quality and quantity of RNA extracted from different tissues were verified on NanoPhotometer (Implen) and 1.5% (w/v) agarose gel electrophoresis. RNA samples (1 μg) were reverse transcribed using SensiFAST cDNA synthesis kit (Bioline) according to manufacturer’s protocol. Afterward, qPCR was performed using Luna Universal qPCR Master Mix (New England Biolabs) with 1 μl of cDNA and 0.25 μM of gene-specific primers (Table S6) to test four genes expression: *NpOMT* encoding norbelladine-*O*-methyltransferase, *NpNBS* encoding norbelladine synthase, *NpNR* encoding noroxomaritidine/norcraugsodine reductase, and *histone3* as endogenous reference endogenous gene. The relative expression of gene was determined by using the 2^−ΔCt^ method.

### Heterologous expression and purification of *Np*OMT

The ORF of full-length *NpOMT* was amplified from *N. papyraceus* bulbs cDNA using PrimeStar GXL premix (TaKaRa Bio) in 50 μl reaction with 0.3 μM of gene-specific primer having BamHI and HindIII in forward and reverse primer. PCR program parameters: 2 min 98 °C for 1 cycle, 10 s 98 °C, 20 s 55 °C, 1 min 72 °C for 35 cycles, 5 min 72 °C for 1 cycle, and final infinite hold at 4 °C. Then amplified region of cDNA was digested with BamHI and HindIII, then cleaned up with GenepHlow Gel/PCR kit (Geneaid). The digested PCR product was cloned into a pMAL-c2X vector by using T_4_ DNA ligase (New England Biolabs). The recombinant plasmids were transformed into chemically competent *E. coli* DH5α cells by heat shock and colonies were selected on LB agar plates containing ampicillin (100 μg/ml, Thermo Fisher Scientific). The further presence of the target gene was confirmed by colony PCR and the identity of the nucleotide was confirmed by sequencing.

For heterologous expression of the *Np*OMT protein, the recombinant plasmid was transformed in *E. coli* Rosetta (DE3) pLysS (EMD Millipore) strain and single colonies were used to inoculate 50 ml of LB medium supplemented with 100 g/ml of ampicillin and 35 g/ml of chloramphenicol. Cultures were grown at 37 °C with orbital shaking at 200 rpm for overnight and used to inoculate 1 L of LB media (100 g/ml of ampicillin and 35 g/ml of chloramphenicol to a starting A_600_ 0.1). Cultures were grown at 37 °C until A_600_ 0.6, cooled on ice for 5 min, and the production of recombinant protein was induced by the addition of IPTG to a final concentration of 0.5 mM. Cultures were kept at 18 °C with shaking at 200 rpm for 16 h, and cells were harvested by centrifugation at 5000*g* for 10 min at 4 °C. Cell pellets were resuspended in 50 ml of protein extraction buffer (30 mM Tris–HCl, pH 8, 150 mM NaCl, 1 mM EDTA, 10% (v/v) glycerol) incubated for 30 min in ice and sonicated on ice for 5 min (10-s on, 10-s off). The crude lysate was centrifuged at 16,000*g* for 30 min at 4 °C to remove cellular debris. The cleared supernatant was incubated with 1 ml of amylose resin beads (New England Biolabs) and incubated at 4 °C with constant shaking. After 1 h, the mixture was centrifuged at 1000g and the supernatant was discarded and washed with 20 ml of washing buffer (same composition of lysis buffer) 2 times. Purified *Np*OMT protein was eluted by using elution buffer (same composition of lysis buffer with the addition of 25 mM of freshly prepared maltose) and subsequently concentrated and desalted by repeated ultrafiltration on an Amicon Ultra 30K column (EMD Millipore) in storage buffer (30 mM Tris–HCl, pH8, 10% (v/v) glycerol). Purified MBP-tagged *Np*OMT concentration was determined using the Bradford reagent according to the manufacturer’s protocol (Thermo Fisher Scientific), with bovine serum albumin as the standard. Protein purity was assessed by 10% (w/v) SDS-PAGE gel.

### Enzymatic assay of *Np*OMT

An initial enzymatic assay of *Np*OMT was performed according to *Kilgore et al.* procedure (21). First, three potential substrates (*i.e.* norbelladine, 3,4-DHBA, and caffeic acid) were screened for the enzymatic assay. Briefly, an enzymatic reaction was carried out in a 50 μl reaction volume containing substrate of interest (norbelladine 0.1 mM, 3,4-DHBA 1 mM, or caffeic acid 1 mM), 2 mM of SAM (methyl donor), 20 μM of purified MBP-tagged *Np*OMT, and 30 mM sodium glycine buffer (pH 9). Reaction mixture was incubated at 37 °C for 1 h. To determine enzyme promiscuity, we incubated the enzyme with ferulic acid, isoferulic acid, vanillin, isovanillin, tyramine (all at 1 mM), 3′-*O*-methylnorbelladine, or 4′-O-methylnorbelladine (both at 0.1 mM). In addition, we performed experiments with *Np*OMT and norbelladine by quenching reaction in 5 min, 15 min, 30 min, 60 min, and 120 min time intervals. The enzymatic reaction was quenched by 100 μl of LC-MS/MS grade methanol, and 1000 mg/L of papaverine was added as internal standard and centrifuged at 12,000 rpm for 5 min. The product(s) formation was carried out by LC-MS/MS.

### HPLC-MS/MS analysis

Targeted metabolite analysis for AAs precursor molecules and AAs were performed on roots, bulb, and leaves tissue. HPLC coupled with MS/MS (Agilent) equipped with an Agilent Jet Stream ionization source, a Kinetex EVO C18 column (150 × 4.6 mm, 5 μm, 100 Å; Phenomenex), a binary pump, an autosampler set at 4 °C, and a column compartment were used for the analyses. Five microliters of each sample were injected into the column that was set at 30 °C. A gradient made of (A) formic acid 0.1% v/v in Milli-Q water and (B) formic acid 0.1% v/v in methanol, with a flow rate of 0.4 ml/min, was used to achieve chromatographic separation. The HPLC elution program started with 10% solvent B; 0 to 10 min, isocratic conditions with 10% B; 10 to 20 min, linear gradient to reach 100% B; 20 to 25 min, isocratic conditions with 100% B; 25 to 26 min, linear gradient to return to initial conditions of 10% B. The total run time was 30 min per sample to allow the reconditioning of the column prior to the next injection. The parameters used for the MS/MS source to perform the analyses were set as follows: gas flow rate 10 L/min, gas temperature 300 °C, nebulizer 45 psi, sheath gas flow 11 L/min, sheath gas temperature 300 °C, capillary voltage 4000 V in ESI+, and nozzle voltage 500 V. Agilent MassHunter Data Acquisition (version 1.2) software (https://www.agilent.com/en/product/software-informatics/mass-spectrometry-software) was used to control the HPLC-MS/MS. MassHunter Qualitative Analysis (version 10.0) and MassHunter Quantitative QQQ Analysis (version 10.0) softwares were used for data processing. Compounds identification were made using authentic standards and analytical parameters presented in Table S4. Internal standard papaverine was added to each sample at a final concentration of 1000 mg/L to allow normalization of each detected signal obtained by HPLC-MS/MS. To obtain accurate relative quantification of targeted metabolites was achieved using area ratios where the peak area of each analyte was divided by the peak area of papaverine.

For enzymatic assays, HPLC-MS/MS analysis were conducted similarly as described previously except the HPLC gradients which were different depending of the analytes to be analyzed in the enzymatic assays. Enzymatic assays performed with 3,4-DHBA and caffeic acid as substrates were analyzed with the following HPLC elution program: 0 min, 35% B; 5.0 min, 50% B; 8.0 min, 60% B; 8.1 min, 35% B. The total run time was 10 min per sample to allow the reconditioning of the column prior to the next injection. Enzymatic assays made with norbelladine as substrate were analyzed with the following gradient: 0 min, 10% B; 10.0 min, 10% B; 20.0 min, 100% B; 25 min, 100% B; 30 min, 90% B. The total run time was 35 min per sample to allow the reconditioning of the column prior to the next injection. Data processing and identifications were made with the same softwares described previously and using authentic standards. Table S5 present analytical acquisition parameters used on HPLC-MS/MS for each enzymatic assays.

### Optimization of *Np*OMT enzymatic reaction and determination of kinetic parameters

To determine the catalytic properties of *Np*OMT, the enzymatic reaction was optimized for temperature and pH range using 3,4-DHBA as substrate. The reaction was carried out at a temperature range of 20 to 60 °C. To determine the optimal pH for *Np*OMT enzymatic reaction, reaction mixture was incubated with sodium citrate buffer (pH 4.5), sodium phosphate buffer (pH 6, 7, and 7.4), Tris–HCl buffer (pH 8), sodium glycine buffer (pH 9), Na_2_HPO_4_-NaOH buffer (pH 11.0), and KCl-NaOH buffer (pH 13.0). The optimal temperature and pH were then selected for further study. The metal dependency of *Np*OMT was determined by using divalent metal ions, such as Mg^+2^, Zn^+2^, Ni^+2^, Co^+2^, and Ca^+2^ at a final concentration of 1 mM in the reaction performed with 1 mM of 3,4-DHBA or 0.1 mM of norbelladine as substrate. Depending upon the effect of metal on the rate of reaction, specific divalent metal ions were selected for further study. Enzyme kinetic parameters of *Np*OMT with 3,4-DHBA were determined with substrate concentration range of 25 to 2000 μM and 5 μM of purified MBP-tagged *Np*OMT at optimized condition. Enzyme kinetic parameters of *Np*OMT with norbelladine were determined with substrate concentration range of 7.62 to 250 μM and 5 μM of purified MBP-tagged *Np*OMT at optimized condition. All analyses were done with LC-MS/MS as described above. Kinetic parameters of *Np*OMT with different substrates were determined by using nonlinear regression from GraphPad Prism 8.0.1.

### Subcellular localization of *Np*OMT and *in vivo* enzymatic assay with norbelladine

For the subcellular localization study, *NpOMT* from *N. papyraceus* was amplified from bulb cDNA. The PCR reaction was performed in 25 μl reaction volume with 200 μM of dNTP, 0.5 μl of high fidelity Q5 DNA polymerase, and 0.2 μM of forward and reverse primers. PCR parameter was 98 °C for 30 sec-1 cycle, 98 °C for 10 s, 72 °C for 30 s, 72 °C for 1 min - 30 cycles, and 72 °C for 2 min. AttB-flanked PCR products of *NpOMT* were purified by using Gel/PCR DNA fragment purification kit (Geneaid). The Gateway cloning BP reaction (Thermo Fisher Scientific) was performed with pDONR221 vector to generate the entry clone which was transformed into *E. coli* DH5α strain and positive clone selected on LB agar plate containing 50 μl of kanamycin. Further sequence identity was confirmed by sequencing. Then LR reaction of entry clone was performed with pB7FWG2 (C-terminal GFP) or pB7WG2F (N-terminal GFP) vector and transformed into *E. coli* DH5α. A positive colony was selected on LB agar plate containing 50 μl of streptomycin. pB7FWG2 or pB7WG2F having full ORF of *NpOMT* was transformed into *Agrobacterium tumefaciens* strain GB3101 by electroporation and a positive colony was selected on LB agar plate containing rifampicin, streptomycin, and gentamycin at 28 °C. Single positive colonies of Agrobacteria harboring pB7FWG2:*Np*OMT or pB7WG2F:*Np*OMT were selected and plated on LB media containing rifampicin, streptomycin, and gentamycin overnight. Agrobacteria cells were harvested by using a sterile loop and washed twice with induction buffer (10 mM MgCl_2_, 10 mM MES buffer pH-5.6, and 200 μM acetosyringone). Then, *A. tumefaciens* culture was diluted to A_600_ 0.5 and incubated in induction buffer for 2 h at room temperature. Finally, *Agrobacterium* culture was infiltrated into 4-weeks old young leaves of *N. benthamiana* plant. Infiltrated *N. benthamiana* plant was cultured for 48 h. For co-expression analysis, pB7FWG2:*Np*OMT or pB7WG2F:*Np*OMT were co-infiltrated with RFP (nucleo-cytosolic marker) and mCherry (with signal peptide to ER marker).

Forty-eight hours post-infiltration, the abaxial epidermis of leaves was placed in a water drop containing DAPI at a final concentration of 1 μg/ml, covered by a slip, and imaged immediately. Images were captured with a Leica TCS SP8 confocal laser scanning microscope (Leica Microsystems) with a 40 × 1.30 oil immersion objective. The GFP excitation wavelength used was 488 nm and the emission of fluorescence signals was detected from 500 to 525 nm. Chlorophyll auto-fluorescence was observed with an excitation wavelength of 552 nm and the emission of fluorescence signals was detected from 630 to 670 nm. The mCherry excitation used was 587 nm and emission of fluorescence signals was 619 nm. The images were first processed in the Las AF Lite software and the combined images were generated using the Las X program from leica microsystem.

To confirm the potential of *Np*OMT to methylate norbelladine in a heterologous *in vivo* system, 50 μl of norbelladine were infiltrated into the *Np*OMT-expressed leaves and incubated for 24 h in a growth chamber. Hundred milligrams of Norbelladine-infiltrated leaves were harvested and homogenized under the liquid nitrogen and then extracted with 100 μl of LC-MS grade methanol. Methanol extraction of *Np*OMT-expressing leaves with norbelladine were centrifuged at 10,000 rpm for 5 min, supernatant was filtered using a 0.2 μm Nylon PTEE filter, and analyzed by LC-MS/MS as described above for enzymatic assays of norbelladine.

### Western blot analysis

To confirm the integrity of GFP-tagged *Np*OMT, we performed the western blotting. A leave disc of 1 cm diameter of GFP-tagged *Np*OMT, GFP alone, and noninfiltrated *N. benthamiana* were harvested and ground under liquid nitrogen. Crude protein was extracted by using 100 μl of protein sample loading buffer and boil at 95 °C for 5 min. Plant debris was sedimented by brief centrifugation, and supernatant was fractionated by using 10% SDS page. Then, migrated proteins were transfered into a polyvinylidene difluoride (PVDF) membrane by using Turbo transfer system (Bio-Rad). Then PVDF membrane was equilibrated with Tris-buffer saline for 5 min, and blocking was done with 10 ml ofTris-buffer saline with 0.02% of tween 20 (TBST) containing 5% of commercial skim milk powder for 1 h at room temperature. PVDF membrane was incubated with TBST containing 3% bovine serum albumin and 1:1000 diluted mouse anti-GFP monoclonal antibody (Cedarlane (CLH106AP)) overnight at 4 °C. Then the membrane was washed five times with TBST, each lasting 5 min and further incubated with TBST containing 5% skim milk and 1:10,000 diluted anti-mouse horse peroxidase antibody. The PVDF membrane was washed six times each for 5 min with TBST and developed clarity ECL substrate and visualized by Bio rad gel doc XR system.

### Protein structure prediction and docking study

The predicted models of norbelladine 4ˈ-*O*-methyltransferase of *Np*OMT and *L. radiata* (*Lr*OMT) were downloaded from the AlphaFold protein database (AF-A0A346TLF5-F1; AF-AOA5BP8H727, respectively). Models were visualized and superimposed using Pymol (Shrödinger). MOE2022.09 software (Chemical Computing Group, www.chemcomp.com/en/Products.htm) was further used to analyze the resulting models conformation and prepare receptors for docking, as described in (14). SAM and Mg^+2^ were positioned into *Np*OMT structure following superimposition of the pocket residues with the crystal structure of *Rattus norvegicus* and *Homo sapiens* COMT (PDB:1H1D (52) and 3A7E, respectively) in MOE.

To optimize the interaction between the enzyme with both SAM and the Mg^2+^ ion, a 250 ns molecular dynamic simulation was performed. The system was constructed using CHARMM-GUI (53, 54, 55). MD simulations were conducted with NAMD software, using the CHARMM36 force field parameters (56, 57). Simulations were carried out at 303.15 K under isothermal-isobaric (NPT) ensemble conditions, with a 2-fs time step and periodic boundary conditions. SAM was parametrized with SwissParam (58). The default simulation parameters provided by CHARMM-GUI, *Solution Builder* were employed, and the protocol for system equilibration was applied.

Following the dynamics completion, the receptor was prepared using *Structure preparation* in MOE, which consists of correcting issues, capping, charging termini, selecting appropriate alternate, and calculating optimal hydrogen position and charges using Protonate 3D. Dummies atoms were added to the receptor using Site Finder, and Quickprep was then applied. This included an energy minimization step with tethered active site residues and fixed SAM and Mg^2+^.

Ligands (3,4-DHBA, norbelladine, caffeic acid, and 4ˈ-*O*-methylnorbelladine) isomeric smiles codes were retrieved from PubChem when available and submitted to the ZINC15 database to download 3D data files (59, 60). Ligands were further prepared in MOE, and all protomers predicted at pH = 7.5 were included as possible ligands. The MMFF94 × force field was used. Receptor docking site included dummy atoms, Mg^+2^ and SAM. Triangle Matcher was used as placement method for 200 poses and tethered induced fit as refinement to perform flexible docking, yielding 10 best poses. Docking poses were analyzed by comparison with crystallized COMT-ligand complex (1H1D, 3A7E), and the first pose (with the best docking score) coherent with crystallized OMT and catalyzed reaction was selected for each ligand. This corresponded to one of the first two best predictions of one of the two most abundant protomers by MOE. PLIP was used to analyze the interactions between ligands and receptor residues (61); the images were further processed using PyMOL (Shrödinger). PyMOL was also used to predict H-bonds between the metal ion and the docked ligand.

## Data availability

All mass spectrometry and raw data are available upon request.

## Supporting information

This article contains supporting information.

## Conflicts of interest

The authors declare that they have no conflicts of interest with the contents of this article.
