# Supplementary material for: Kinetic and in silico structural characterization of norbelladine O-methyltransferase of Amaryllidaceae alkaloids biosynthesis
Source: J Biol Chem. 2024 Aug 8;300(9):107649. doi: 10.1016/j.jbc.2024.107649 (PMC11407090; doi:10.1016/j.jbc.2024.107649)
Supplement: Supporting Tables and Figures [file mmc1.docx]

# Kinetic and *in silico* structural characterization of norbelladine *O*-methyltransferase of Amaryllidaceae alkaloids biosynthesis

Running title: Kinetic & In silico study of Norbelladine OMT in alkaloid biosynthesis.

**Supporting information**

Manoj Koirala^1^, Natacha Merindol^1^, Vahid Karimzadegan^1 ,^Sarah-Eve Gélinas^1^, Nuwan Sameera Liyanage^1^, , Basanta Lamichhane^1^, Maria Camila García Tobón^1^, Patrick Lagüe^2^, Isabel Desgagné-Penix ^1,3*^

^1^Department of Chemistry, Biochemistry and Physics, Université du Québec à Trois-Rivières, Trois-Rivières, Québec, Canada

^2^ Department of Biochemistry, Microbiology and Bioinformatics, Laval University, Québec, Canada

^3^Plant Biology Research Group, Trois-Rivières, Québec, Canada

*Corresponding author’s email address: Isabel.Desgagne-Penix@uqtr.ca

**Table S1. The accession number of *O*-methyltransferase for phylogenetic analysis**

| **Name of organism** | **Type of *O*-methyltransferase** | **Class of *O*-methyltransferase** | **Accession number** |
| --- | --- | --- | --- |
| *N. papyraceus* | AAOMT | I | MF979869 |
| *N. sp. Aff. Pseudonarcissus* | AAOMT | I | AIL54541.1 |
| *Lycoris radiata* | AAOMT | I | QFQ50503 |
| *Vanilla planifolia* | CCoAOMT | I | AAC49913.1 |
| *Medicago sativa* | CCoAOMT | I | AAC28973.1 |
| *Morus notabilis* | CCoAOMT | I | EXC17316.1 |
| *Nicotiana tabacum* | CCoAOMT | I | AAC49913.1 |
| *Populus tomentosa* | CCoAOMT | I | ACE95173.1 |
| *Plagiochasma appendiculatum* | CCoAOMT | I | KU578317.1 |
| *Danio rerio* | COMT | I | NP-001025328.2 |
| *Egretta garzetta* | COMT | I | KFP16811.1 |
| *Equus caballus* | COMT | I | NP-001108007.1 |
| *Homo sapiens* | COMT | I | NP-000745.1 |
| *Canis lupus* | COMT | I | NP_001004074.1 |
| *Rattus norvegicus* | COMT | I | 1JR4-A |
| *Medicago truncatula* | CaMT | II | DQ419914 |
| *Sorghum bicolor* | CaMT | II | AAL57301 |
| *Arabidopsis thaliana* | CaMT | II | NP-200227 |
| *Stylosanthes humilis* | CaMT | II | 2119166A |
| *Populus tomentosa* | CaMT | II | AAF63200 |
| *Medicago sativa* | CaMT | II | AAB46623.1 |
| *Catharanthus rosesus* | BIAOMT | II | ABR20103.1 |
| *Coptis japonica* | BIAOMT | II | BAB08005.1 |
| *Papaver sonmiferum* | BIAOMT | II | ACN88562.1 |

Abbreviation: AAOMT: Amaryllidaceae Alkaloid *O*-methyltransferase, CCoAOMT: caffeoyl CoA *O*-methyltransferase, COMT: catecol *O*-methyltransferase, CaOMT: caffeic acid *O*-methyltransferase and BIAOMT: Benzylisoquinoline *O*-methyltransferase

**Table S2. Identity and similarity (%) between characterized norbelladine *O*-methyltransferase and *Np*OMT.**

|  | **IDENTITY** | | | | | |
| --- | --- | --- | --- | --- | --- | --- |
| **SIMILARITY** |  | ***Np*OMT** | ***Np*N4OMT** | ***Lr*OMT** | ***La*OMT** | ***Ge*OMT** |
|  | ***Np*OMT** | - | 95.40 | 92.05 | 91.63 | 92.89 |
|  | ***Np*N4OMT** | 98.74 | - | 90.79 | 90.38 | 92.05 |
|  | ***Lr*OMT** | 97.49 | 96.23 | - | 97.07 | 92.47 |
|  | ***La*OMT** | 97.49 | 96.23 | 98.33 | - | 91.63 |
|  | ***Ge*OMT** | 96.23 | 95.82 | 96.23 | 95.40 | - |

**Table S3. Differences in residues between enzymes of different substrate specificity.**

| Position | 3’- and 4’-*O*-methy-lation of norbelladine | only 4’-*O*-methylation of norbelladine | 4’-*O*-methylation and demethylation of norbelladine | 3,4-DHBA or caffeic acid 3 or 4-*O*-methylation |
| --- | --- | --- | --- | --- |
| 9 | A/S | S | A | A/S |
| 20 | D | D | E | E/D |
| 30 | V | A/T | A | A/T/V |
| 108 | Y | Y/F | F | Y/F |
| 140 | L | V/L | V | V/L |
| 141 | Q | E/R | Q | Q/E/R |
| 148 | F | L/F | L | L/F |
| 151 | F | Y/F | Y | Y/F |
| 179 | V/I | L/A | L | L/A/V/I |
| 186 | F | Y | Y | F/Y |
| 188 | T | S | S | T/S |
| 191 | F | Y | Y | F/Y |
| 203 | E | V/E | V | V/E |
| 204 | C | A/C | A | A/C |
| 206 | V | L | L | V/L |
| 210 | N | N | Q | Q/N |
| 213 | K | T | T | K/T |
| 228 | V/T | I/V | I | I/V/T |
| 232 | L | V | V | V/L |

**Table S4. *Np*OMT catalytic site residues interacting with docked ligands, metal ion, and SAM.**

| Active Site residues | Ligand | Score | Interactions | | |
| --- | --- | --- | --- | --- | --- |
|  |  |  | **Hydrophobic** | **H-bonds** | **Other** |
| Asp6, Asp7, Tyr8, Ala9, Leu10, Ile11, His12, Lys13, Glu46, Lys47, His48, Glu49, Trp50, Ser51, Ser52, Ala53, Ile78, Gly79, Val80, Tyr81, Ile102, Asp103, Val104, Asn105, Phe108, Ser130, Glu131, Ala132, Leu133, Pro134, Asp155, Ala156, Asp157, Lys158, Asn160, Tyr164, Asp181, Asn182, Trp185, Tyr186, Ser188, Tyr194, Leu197, His198, Glu200, Glu201, Ala204, Asp230, | Mg^2+^ | na | Nd | Nd | Metal ion: Asp155 (n=2), Asp181, Asn182 |
|  | Mg^2+^ 250 ns | na | Nd | Nd | Metal ion:  Asp155 (n=2), Asp181 (n=2),  Asn182 |
|  | SAM | na | Tyr81 | Val55, Tyr81, Tyr84, Ser85, Asp103, (n=2), Val104, Ala132, Asp155, Asp157 (n=2) | Salt bridge: Asp155 |
|  | SAM 250 ns | na | Tyr81 | Val55 (2.48), Val80 (3.08 Å), Tyr81(2.98 Å ), Thr82 (3.22 Å), Gly83 (2.37 Å), Tyr84 (2.25 Å), Ser85, (2.26 Å) Asp103, (n=2; 2.28 Å, 1.91 Å), Val104 (3.03 Å), Ala132 (2.37 Å), Asp155, Tyr164 (3.07 Å) | Salt bridge: Asp155 |
|  | Norbelladine to 3ˈ-*O*-methylnorbelladine | nd | na | na | na |
|  | Norbelladine to 4ˈ-*O*-methylnorbelladine | -6.26 | Lys13, Trp185 (n=2) | Lys13 (2.43 Å), Ser52 (n=2; 2.83 Å, 2.26 Å), Leu54 (3.03 Å), Lys158 (2.68 Å), Asn182 (2.16 Å) | π−stacking Tyr186;  Metal ion:  Mg^2+^ |
|  | 4ˈ-*O*-methylnorbelladine to 3ˈ4ˈ-*O*-dimethylnorbelladine | -5,42 | Ly158, Glu200 | Lys158 (2.39 Å), Asn182 (2.31 Å), Ala204 (3.65 Å) | Metal ion: Mg^2+^ |
|  | 3,4-DHBA to isovanillin (4-*O*) | -4,56 | Trp185 | Lys13 (2.82 Å), Ser52 (n=2, 2.43 Å, 3.62 Å), Lys158 (2.46 Å), Asn182 (2.14 Å) | Metal ion:  Mg^2+^ (n=2) |
|  | 3,4-DHBA to vanillin (3-*O*) | -4,19 | nd | Ly13 (3.11 Å), Ser52 (2.24 Å), Lys158 (2.45 Å), Asn182 (2.16 Å) | π−stacking Trp185 Metal ion:  Mg^2+^ (n=2) |
|  | Caffeic acid to  isoferulic acid (4-*O*) | nd | na | na | na |
|  | Caffeic acid to  ferulic acid (3-*O*) | -4.70 | Lys158, Trp185 | Lys13 (3.30 Å), Ser52 (1.93 Å), Lys158 (3.03 Å), Asn182 (2.11 Å) | Salt bridge Lys158  Metal ion: Mg^2+^ |

Na: not applicable, nd: not detected. The score is in (kCal/mol)

**Table S5. MRM transitions and instrumental parameters used for HPLC-MS/MS analyses in ESI+ and ESI-.** The quantifier product ion for each compound is written in bold and qualifier product ions are not.

| **Compound name** | **Retention time (min)** | **Precursor (*m/z*)** | **Product (*m/z*)** | **Fragment (V)** | **CE**  **(V)** | **Polarity (ESI +/-)** |
| --- | --- | --- | --- | --- | --- | --- |
| 3,4-Dihydroxybenzaldehyde | 15.795 | 137 | 119 | 114 | 20 | Negative |
|  |  |  | **108** |  | 26 |  |
|  |  |  | 92 |  | 26 |  |
| 3'-*O*-Methylnorbelladine | 16.875 | 274 | **137** | 105 | 18 | Positive |
|  |  |  | 121 |  | 15 |  |
|  |  |  | 109 |  | 40 |  |
| 4'-*O*-Methylnorbelladine | 15.630 | 274 | **137** | 105 | 18 | Positive |
|  |  |  | 122 |  | 40 |  |
|  |  |  | 94 |  | 40 |  |
| 11-Hydroxyvittatine | 4.529 | 288 | 226 | 105 | 26 | Positive |
|  |  |  | **196** |  | 34 |  |
|  |  |  | 153 |  | 40 |  |
| 3',4'-*O*-Dimethylnorbelladine | 16.668 | 288 | **151** | 100 | 10 | Positive |
|  |  |  | 135 |  | 40 |  |
|  |  |  | 107 |  | 40 |  |
| Caffeic acid | 18.344 | 181 | **163** | 76 | 5 | Positive |
|  |  |  | 135 |  | 10 |  |
|  |  |  | 117 |  | 21 |  |
| Ferulic acid | 19.588 | 195 | **177** | 81 | 5 | Positive |
|  |  |  | 145 |  | 20 |  |
|  |  |  | 117 |  | 21 |  |
| Galanthamine | 4.655 | 288 | 270 | 120 | 14 | Positive |
|  |  |  | 225 |  | 18 |  |
|  |  |  | **213** |  | 22 |  |
| Haemanthamine | 12.204 | 302 | 211 | 105 | 22 | Positive |
|  |  |  | **196** |  | 34 |  |
|  |  |  | 181 |  | 30 |  |
| Homolycorine | 15.892 | 316 | 300 | 115 | 30 | Positive |
|  |  |  | **298** |  | 22 |  |
|  |  |  | 94 |  | 26 |  |
| Isoferulic acid | 19.762 | 195 | **177** | 81 | 5 | Positive |
|  |  |  | 145 |  | 20 |  |
|  |  |  | 117 |  | 21 |  |
| Isovanillin | 18.566 | 153 | 125 | 86 | 10 | Positive |
|  |  |  | **93** |  | 13 |  |
|  |  |  | 65 |  | 25 |  |
| Lycorine | 3.963 | 288 | 270 | 92 | 20 | Positive |
|  |  |  | 177 |  | 20 |  |
|  |  |  | **147** |  | 28 |  |
|  |  |  | 119 |  | 40 |  |
| Narciclasine | 18.367 | 308 | **248** | 82 | 28 | Positive |
|  |  |  | 218 |  | 36 |  |
|  |  |  | 202 |  | 40 |  |
| Norbelladine | 7.181 | 260 | **138** | 86 | 5 | Positive |
|  |  |  | 123 |  | 20 |  |
|  |  |  | 121 |  | 17 |  |
| Pancracine | 6.468 | 288 | **270** | 95 | 18 | Positive |
|  |  |  | 185 |  | 34 |  |
|  |  |  | 165 |  | 40 |  |
| Papaverine | 18.004 | 340 | 324 | 165 | 40 | Positive |
|  |  |  | **202** |  | 27 |  |
|  |  |  | 171 |  | 40 |  |
| *p*-Coumaric acid | 19.448 | 165 | **147** | 76 | 5 | Positive |
|  |  |  | 119 |  | 17 |  |
|  |  |  | 91 |  | 20 |  |
| Tazzetine | 17.216 | 332 | **314** | 90 | 14 | Positive |
|  |  |  | 181 |  | 30 |  |
|  |  |  | 153 |  | 40 |  |
| *trans*-Cinnamic acid | 21.296 | 147 | **103** | 65 | 9 | Negative |
|  |  |  | 77 |  | 20 |  |
| Vanillin | 18.740 | 153 | 125 | 86 | 10 | Positive |
|  |  |  | **93** |  | 13 |  |
|  |  |  | 65 |  | 25 |  |
| Crinine | 10.113 | 272 | 226 | 105 | 26 | Positive |
|  |  |  | 196 |  | 34 |  |
|  |  |  | **136** |  | 26 |  |

**Table S6. List of primers used in this study.**

| **Primer code** | **Sequence (5′-3′)** | **Direction** | **Application** |
| --- | --- | --- | --- |
| *Np*OMT-Forward | AACGGGATCCATGGGTGCTAGCCAAGATGAT | Forward | Cloning into pMAlc2x vector |
| *Np*OMT-Reverse | ACGCAAGCTTTCAATAAAGACGTCGGCA | Reverse |  |
| Loc-N-*Np*OMT-For | GGGGACAAGT TTGTACAAAAAAGCAGGCA  TG GGTGCTAGC CAAGATGAT | Forward | N-terminal localization |
| Loc-N-*Np*OMT-Rev | GGGGACCACTTTGTACAAGAAAGCTGGGTC  TCAATAAAGACGTCGACA | Reverse |  |
| Loc-C-*Np*OMT-F | GGGGACAAGTTTGTACAAAAAAGCAGGCTT  CGAAGGAGATAGAACCATGGGTGCTAGCCA  AGATGAT | Forward | C-terminal localization |
| Loc-C-*Np*OMT-R | GGGGACCACTTTGTACAAGAAAGCTGGGTTT  TATCAATAAAGACGTCGGCAAATAGT | Reverse |  |
| qPCR-*Np*OMT-F | CCACGAGCGATTAGTGAAGC | Forward | RT-q-PCR analysis |
| qPCR-*Np*OMT-R | GGGATATCTCGACACGGG GA | Reverse |  |
| qPCR-NBS-F | GAGGGAGGGCACTTG G | Forward |  |
| qPCR-NBS-R | AGGAAGCATTTGCYGCATGT | Reverse |  |
| qPCR-NR-F | AACAATGCAGGGACAGCCAT | Forward |  |
| qPCR-NR-R | GCCGATTCGAAATTGGTGGC | Reverse |  |
| *Np*Histone-F | GTCTGCCCCAACAACTGGAGG | Forward |  |
| *Np*Histone-R | GCTTCCTAATCAGTAGCTCG | Reverse |  |

**
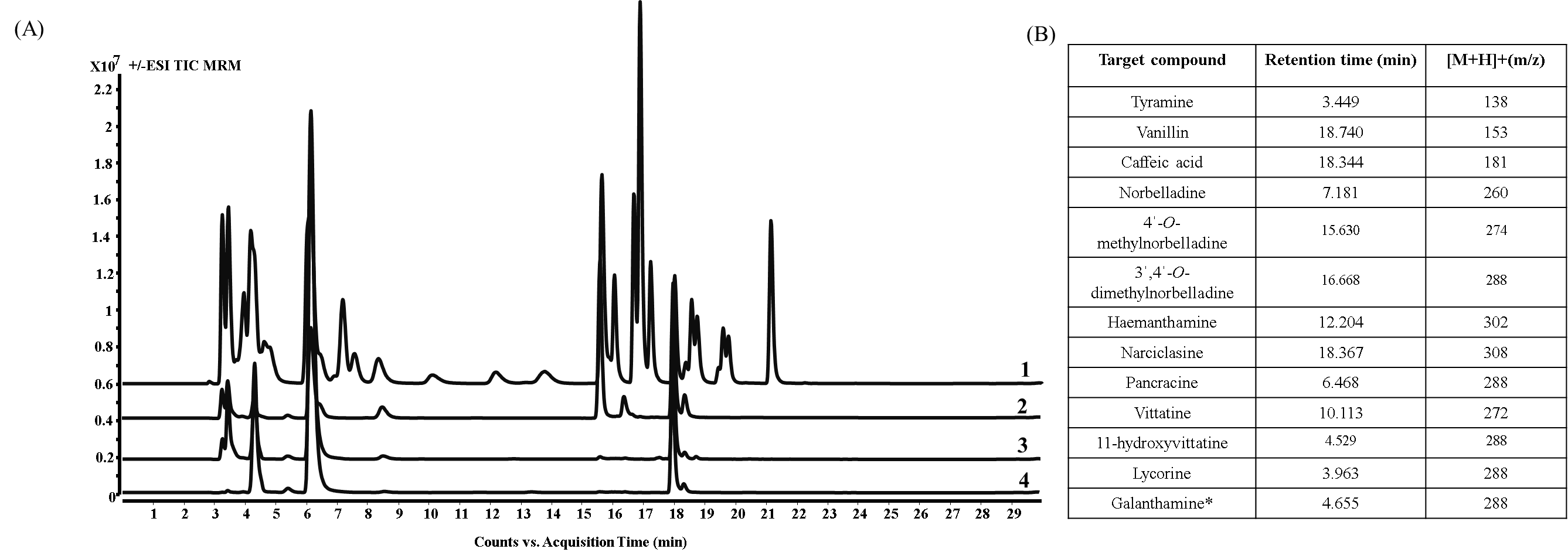
**

**Fig. S1.** **Targeted metabolites analysis on *N. papyraceus* during the vegetative stage**. (A) LC-MS/MS chromatography (1) standard mixture, (2) leaves, (3) bulb, and (4) roots of *Narcissus. papyraceus*. (B) Retention time (min) and M+H +(m/z) mass of identified compounds. * Indicates the low-level detection of the compound.

**
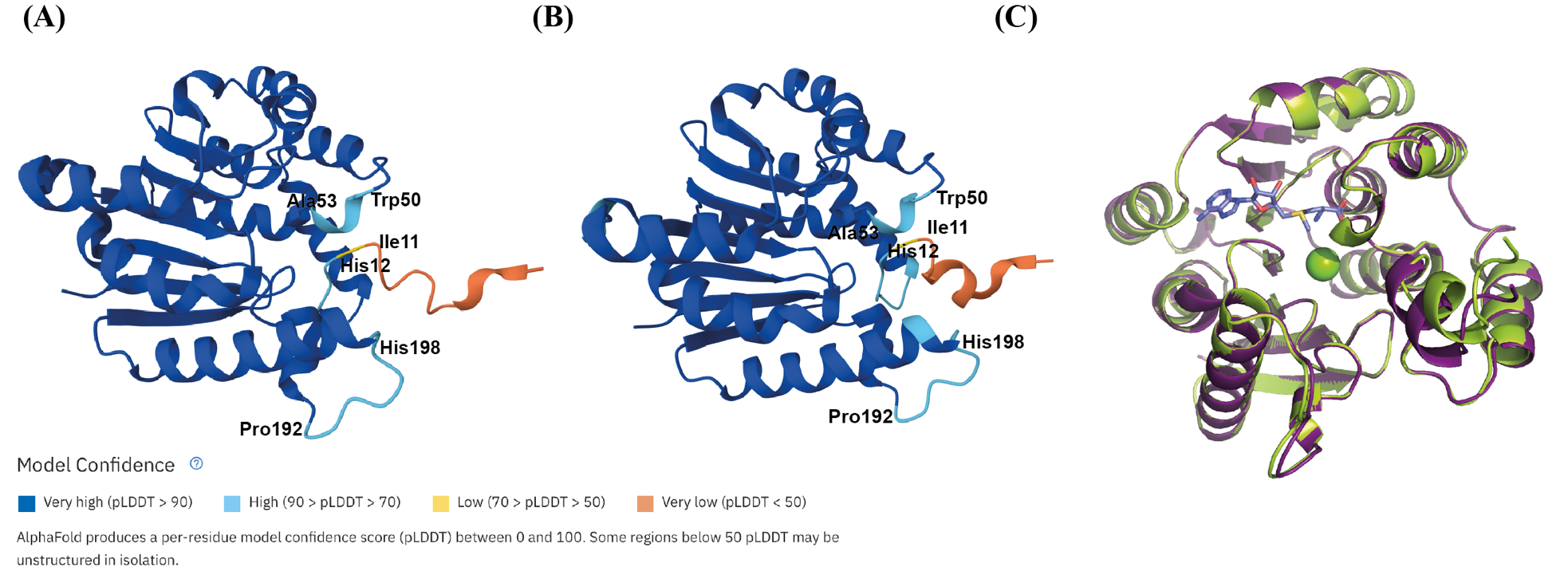
**

**Fig S2. *Np*OMT and *Lr*OMT predicted structure.** (A) Confidence range of the predicted structure of *Np*OMT. (B) Confidence range of the predicted structure of *Lr*OMT. (C) Superposition between the two structures with Mg^2+^ and SAM positioned from PDB 1H1D (RMSD = 0.074).


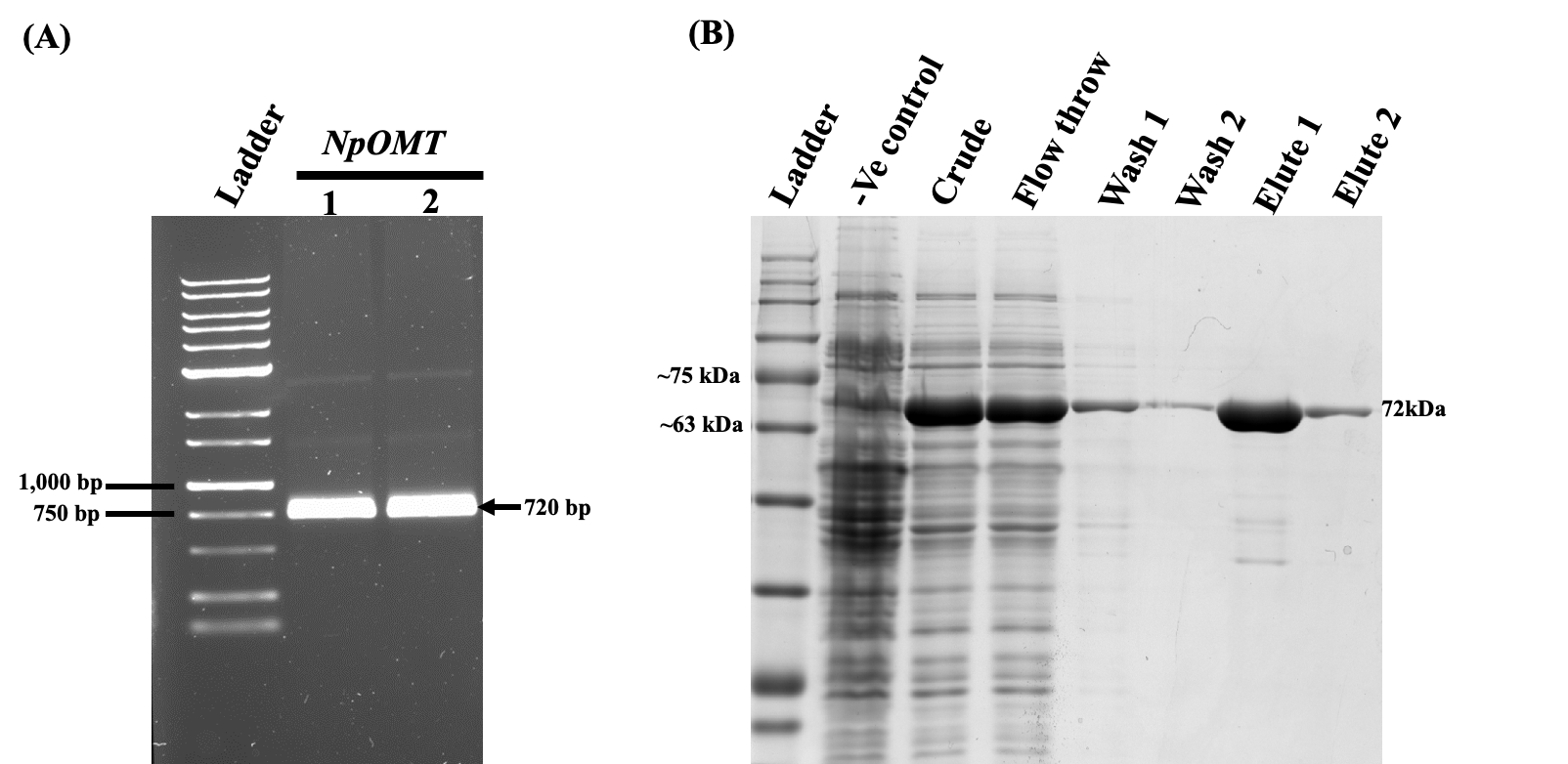


**Fig. S3. Heterologous expression of *Np*OMT in *E. coli* and purification of *Np*OMT.** (A). PCR amplification of *Np*OMT from cDNA of *N. papyraceus*. Lane 1 and 2 shows amplified *NpOMT*. (B) SDS-PAGE analyses of overexpression and purification of recombinant *Np*OMT.


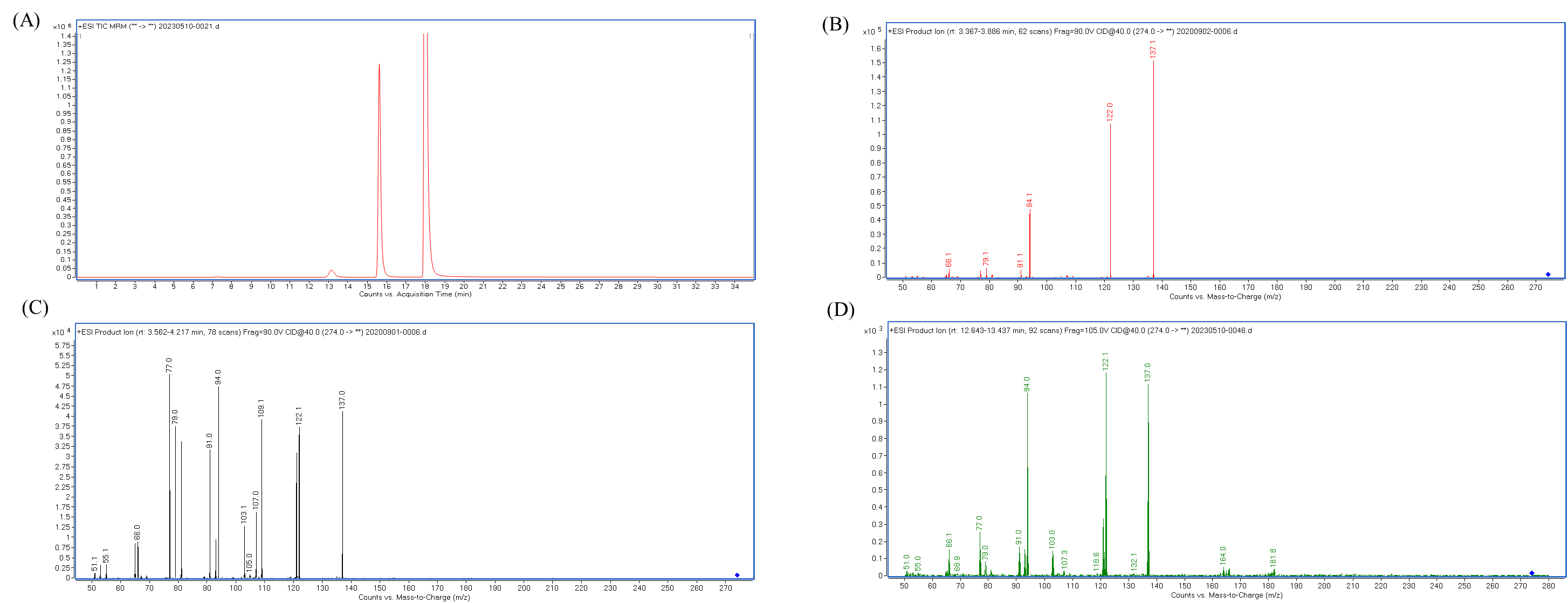


**Fig. S4. LC-MS/MS analysis of peak iv corresponding to fig. 4 (B).** (A) +ESI TCI MRM of *Np*OMT enzymatic reaction with *Np*OMT. (B) Fragmentation of peak (unknown) obtain at 13.1 min, (C) Fragmentation of standard 4ˈ-*O*-methylnorbelladine. and (D) Fragmentation of standard 3 ˈ-*O*-methylnorbelladine.

**
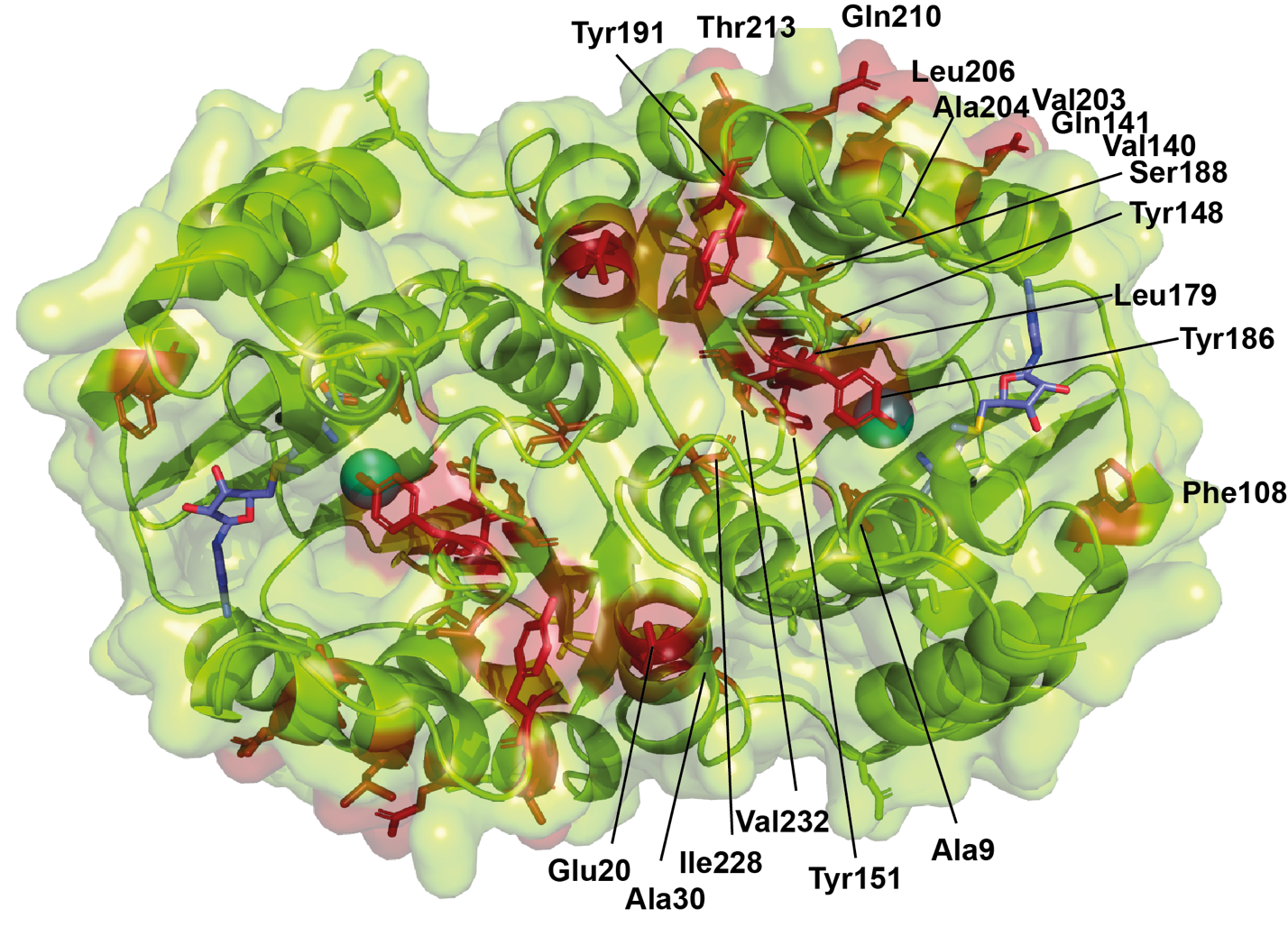
Fig. S5.** **Residues possibly implicated in regioselectivity and substrate specificity.** *Np*OMT homodimer is shown as green cartoon with transparent surface. The 19 identified residues (from Table S3) are shown as red sticks. SAM is displayed as purple sticks and Mg^2+^ as blue spheres.

**
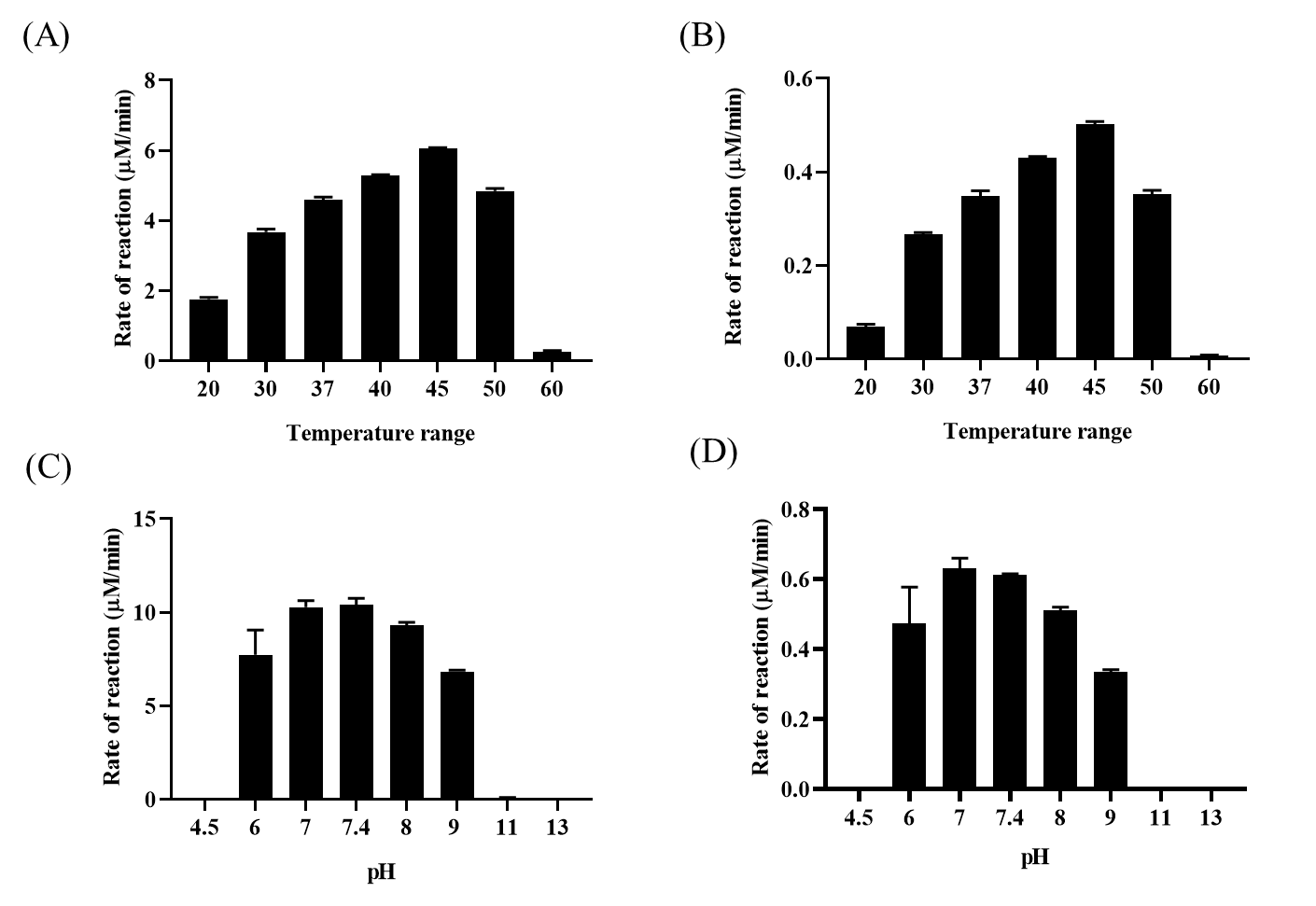
**

**Fig. S6. Optimization pH and temperature for enzymatic reaction of *Np*OMT with 3,4-DHBA**. (A) Effect of temperature on the formation of vanillin. (B) Effect of temperature on the formation of isovanillin. (C) Effect of pH on the formation of vanillin. (D) Effect of pH on the formation of isovanillin. All experimental values represent the means of three replicates ± standard deviation.


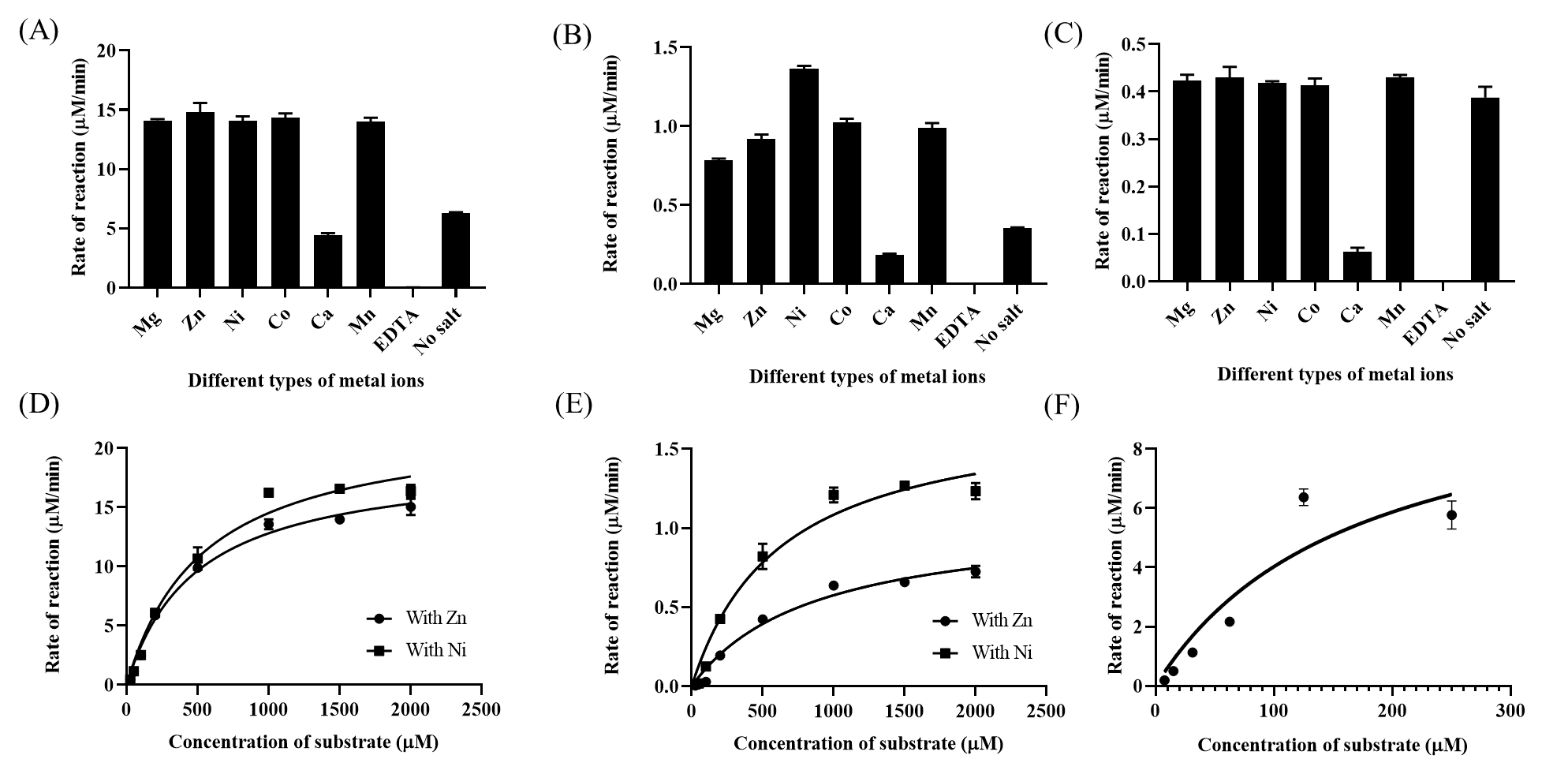


**Fig. S7. Biochemical characterization of *Np*OMT.** Effect of different divalent metal ions on formation of (A) vanillin, (B) isovanillin, and (C) 4ˈ-*O*-methylnorbelladine. The steady-state kinetic parameters for *Np*OMT catalyzed reaction for the formation of (D) vanillin, (E) isovanillin, and (F) 4ˈ-*O*-methylnorbelladine. All experimental values represent the means of three replicates ± standard deviation.

**
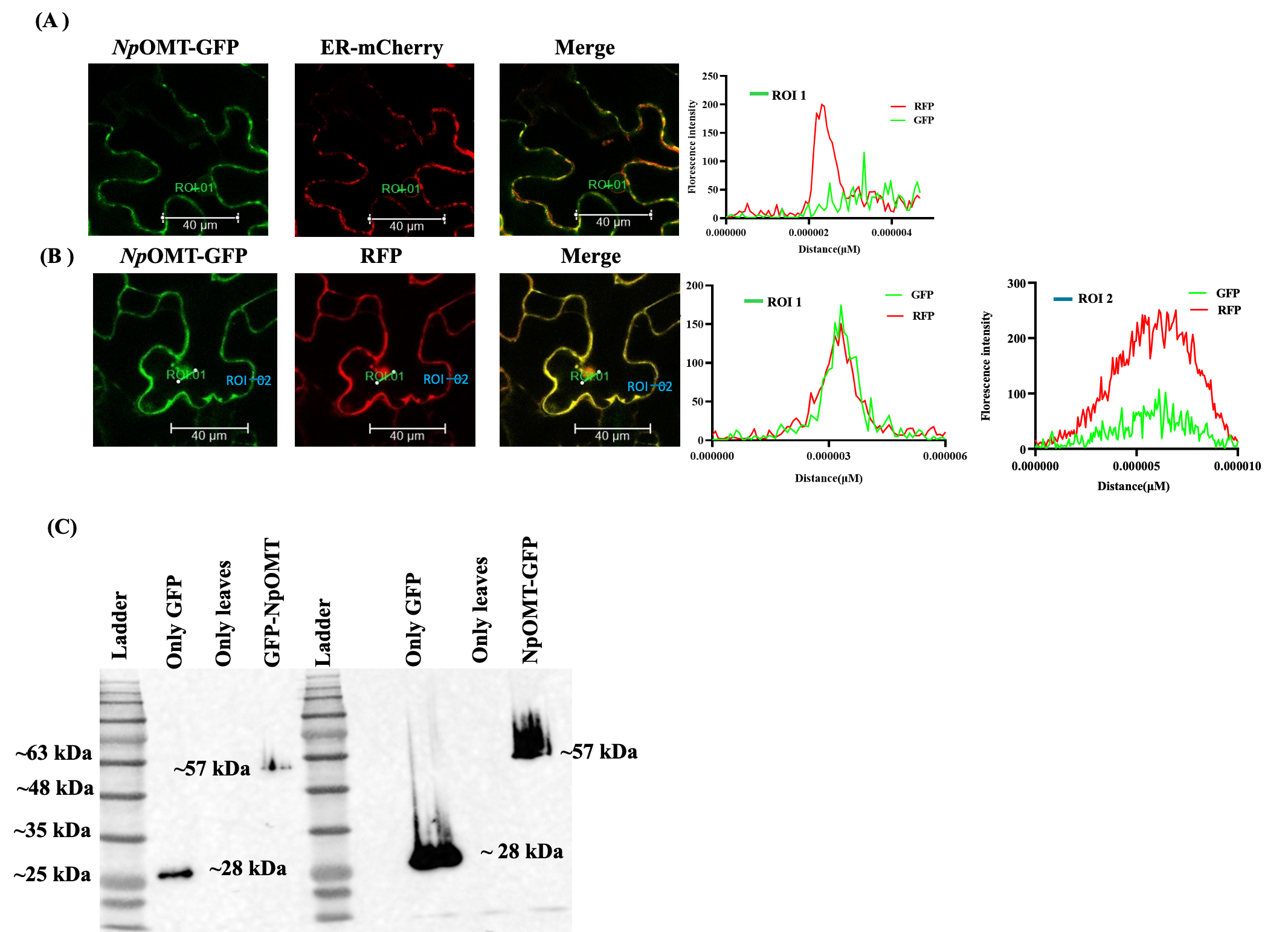
**

**Fig. S8. Cellular location of C-terminal GFP tagged *Np*OMT and confirmation of the integrity of GFP-tag *Np*OMT.** (A) GFP-*Np*OMT, ER-mCherry, merge image and graphical intensity of fluorescent intensity are shown. (B) GFP-*Np*OMT, RFP, merge image, and graphical intensity of fluorescent intensity are shown. The scale bar represents 40 µm. ROI 1 and ROI 2 indicate fluorescent intensity in the nucleus and cytosol, respectively. (C) Western blotting of only GFP (~28 kDa), only leaves, GFP-*Np*OMT (N-terminal GFP tagged *Np*OMT), and *Np*OMT-GFP (C-terminal tagged *Np*OMT). Predicted-size GFP tagged *Np*OMT was ~57 (28 + 29 ) kDa.


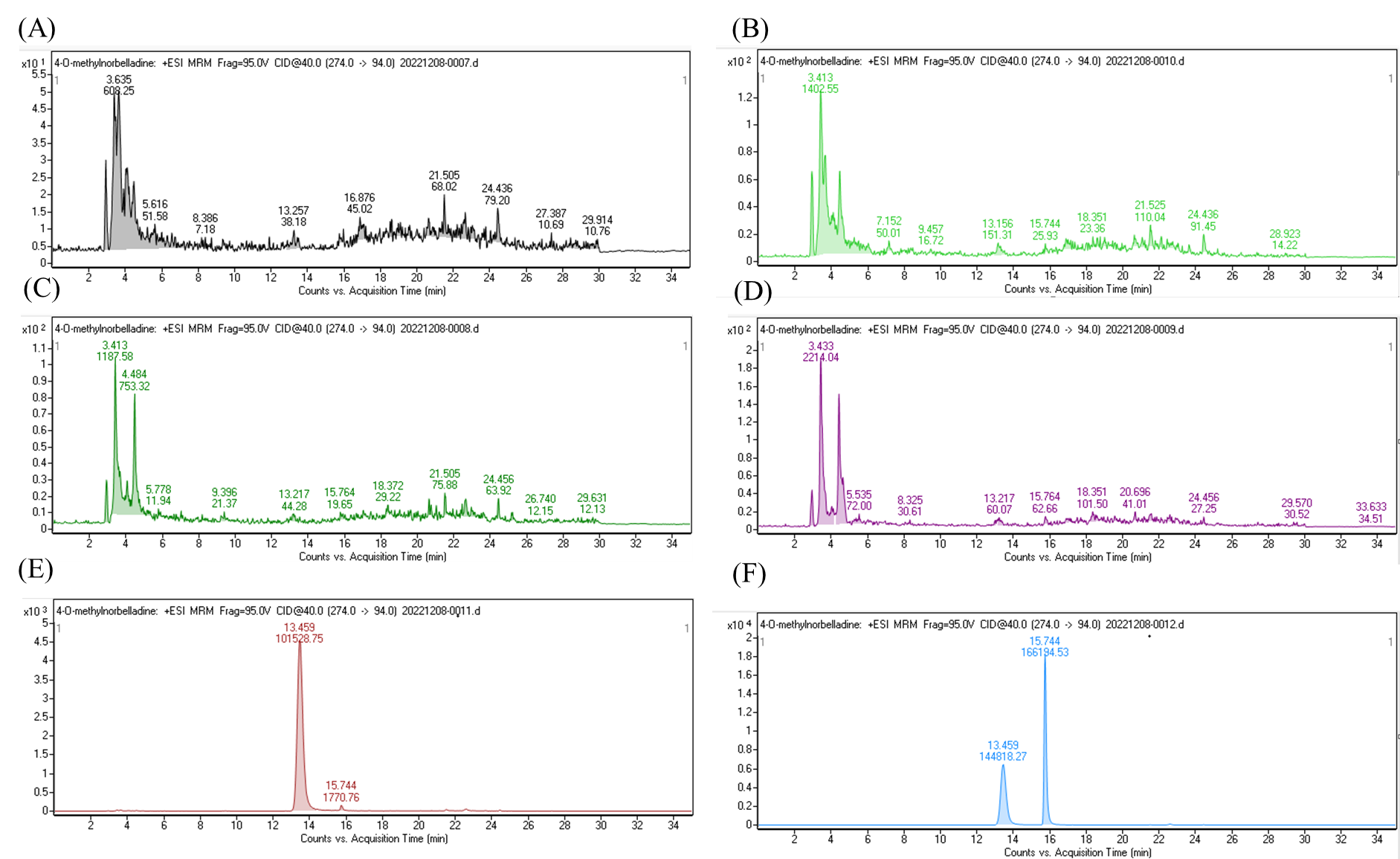


**Fig. S9. Enzymatic assay of *Np*OMT with norbelladine in *Nicotiana benthamiana***. The enzymatic reaction was done with N-terminal GFP tag *Np*OMT and 50 µM norbelladine was infiltrated. The formation of products was monitored was confirmed by LC-MS/MS analysis of methanolic extraction of norbelladine infiltrated leaves after 24 hours. (A) LC-MS/MS analysis of only leaves with transit expression of *Np*OMT, (B) LC-MS/MS analysis of transit expression of *Np*OMT infiltrated with methanol, (C) LC-MS/MS analysis of GFP expressed leaves, (D) LC-MS/MS analysis of N-terminal GFP tag *Np*OMT expressed leaves, (D) LC-MS/MS analysis of norbelladine infiltrated leaves with GFP expressed and (E) LC-MS/MS analysis of norbelladine infiltrated leaves with N-terminal GFP tag *Np*OMT expressed leaves.

**
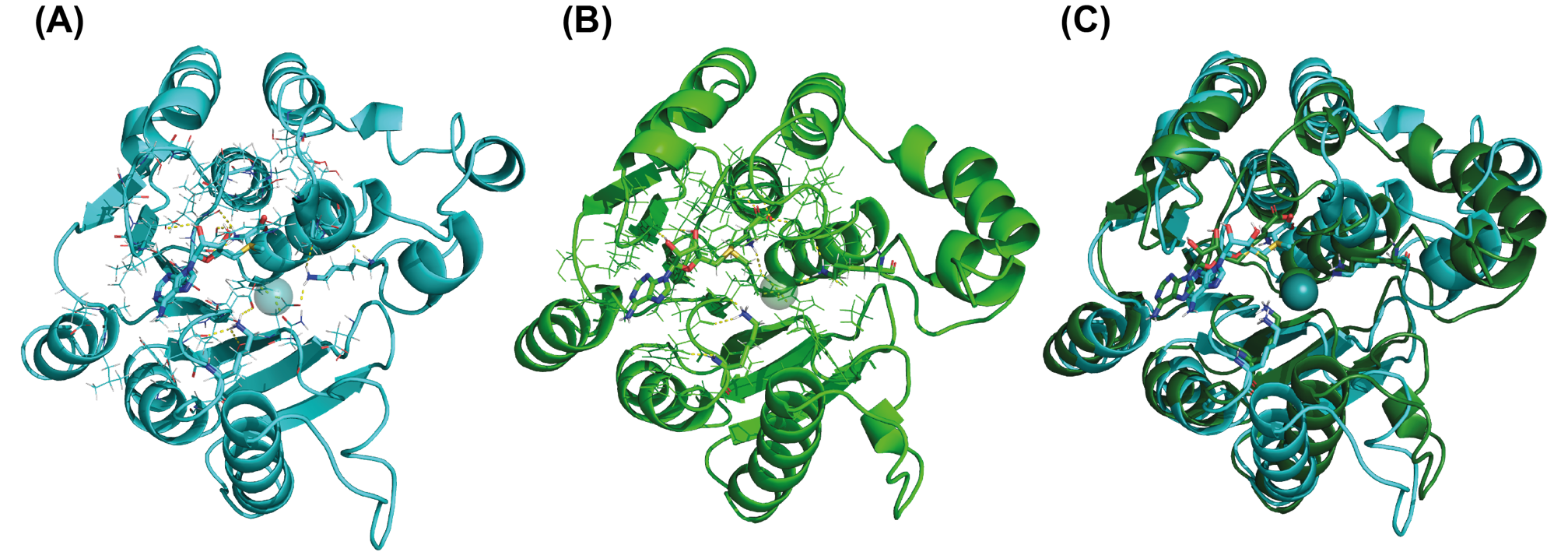
**

**Fig. S10. Changes in conformation of *Np*OMT after a 250 ns trajectory.** (A) Ribbon representation of AlphaFold2 predicted structure of *Np*OMT with lines for the active site residues, sticks for Lys13, Lys158, and SAM and sphere for Mg^2+^. (B) Ribbon representation of *Np*OMT following a 250 ns trajectory, with lines for the active site residues, sticks for Lys13, Lys158, and SAM and sphere for Mg^2+^. H-bonds between Lys13, Lys158 and Mg2+ and the active site residues are shown as dashed yellow lines. (C) Superimposition of *Np*OMT before (turquoise) and following a 250 ns trajectory (green), with sticks for Lys13, Lys158, and SAM and sphere for Mg^2+^.
